# Genome Sequence of the Plant Growth Promoting Endophytic Bacterium *Enterobacter* sp. 638

**DOI:** 10.1371/journal.pgen.1000943

**Published:** 2010-05-13

**Authors:** Safiyh Taghavi, Daniel van der Lelie, Adam Hoffman, Yian-Biao Zhang, Michael D. Walla, Jaco Vangronsveld, Lee Newman, Sébastien Monchy

**Affiliations:** 1 Biology Department, Brookhaven National Laboratory, Upton, New York, United States of America; 2 Mass Spectrometer Center, Department of Chemistry and Biochemistry, University of South Carolina, Columbia, South Carolina, United States of America; 3 Department of Environmental Biology, Universiteit Hasselt, Diepenbeek, Belgium; Stanford University, United States of America

## Abstract

*Enterobacter* sp. 638 is an endophytic plant growth promoting gamma-proteobacterium that was isolated from the stem of *poplar* (*Populus trichocarpa*×*deltoides* cv. H11-11), a potentially important biofuel feed stock plant. The *Enterobacter* sp. 638 genome sequence reveals the presence of a 4,518,712 bp chromosome and a 157,749 bp plasmid (pENT638-1). Genome annotation and comparative genomics allowed the identification of an extended set of genes specific to the plant niche adaptation of this bacterium. This includes genes that code for putative proteins involved in survival in the rhizosphere (to cope with oxidative stress or uptake of nutrients released by plant roots), root adhesion (pili, adhesion, hemagglutinin, cellulose biosynthesis), colonization/establishment inside the plant (chemiotaxis, flagella, cellobiose phosphorylase), plant protection against fungal and bacterial infections (siderophore production and synthesis of the antimicrobial compounds 4-hydroxybenzoate and 2-phenylethanol), and improved poplar growth and development through the production of the phytohormones indole acetic acid, acetoin, and 2,3-butanediol. Metabolite analysis confirmed by quantitative RT–PCR showed that, the production of acetoin and 2,3-butanediol is induced by the presence of sucrose in the growth medium. Interestingly, both the genetic determinants required for sucrose metabolism and the synthesis of acetoin and 2,3-butanediol are clustered on a genomic island. These findings point to a close interaction between *Enterobacter* sp. 638 and its poplar host, where the availability of sucrose, a major plant sugar, affects the synthesis of plant growth promoting phytohormones by the endophytic bacterium. The availability of the genome sequence, combined with metabolome and transcriptome analysis, will provide a better understanding of the synergistic interactions between poplar and its growth promoting endophyte *Enterobacter* sp. 638. This information can be further exploited to improve establishment and sustainable production of poplar as an energy feedstock on marginal, non-agricultural soils using endophytic bacteria as growth promoting agents.

## Author Summary

Poplar is considered as the model tree species for the production of lignocellulosic biomass destined for biofuel production. The plant growth promoting endophytic bacterium *Enterobacter* sp. 638 can improve the growth of poplar on marginal soils by as much as 40%. This prompted us to sequence the genome of this strain and, via comparative genomics, identify functions essential for the successful colonization and endophytic association with its poplar host. Analysis of the genome sequence, combined with metabolite analysis and quantitative PCR, pointed to a remarkable interaction between *Enterobacter* sp. 638 and its poplar host with the endophyte responsible for the production of a phytohormone, and a precursor for another that poplar is unable to synthesize, and where the production of the plant growth promoting compounds depended on the presence of plant synthesized compounds, such as sucrose, in the growth medium. Our results provide the basis to better understanding the synergistic interactions between poplar and *Enterobacter* sp. 638. This information can be further exploited to improve establishment and sustainable production of poplar on marginal, non-agricultural soils using endophytic bacteria such as *Enterobacter* sp. 638 as growth promoting agents.

## Introduction

Endophytic bacteria reside inside living plant tissue without harming it [1]. Endophytic colonization is considered as a sign of a healthy plant system, as many endophytes will promote the growth, health and development of their host plant. Any organ of the plant can by colonized with a broad diversity of bacterial endophytes, many of which are closely related to common soil bacteria representative of genera such as *Enterobacter*, *Pseudomonas*, *Burkholderia*, *Bacillus*, and *Azospirillum* [2]–[4]. The diversity of endophytes is dependent on plant species, cultivar and probably cultivation conditions [5], [6].

Plant roots are the main site of endophytic colonization. Root colonization by bacteria was described to involve several stages [7]. In the initial step bacteria move towards the plant roots either passively via soil water fluxes, or actively via specific induction of flagellar activity by plant-released compounds (chemotaxis). Second, a non-specific adsorption of bacteria to roots occurs, followed by anchoring (3rd step) that results in firm attachment of bacteria to the root surface. Specific or complex interactions between the bacterium and the host plant, such as the secretion of root exudates, may arise resulting in the induction of bacterial gene expression. Finally, endophytic bacteria can enter the plant at sites of tissue damage, which naturally arise as the result of plant growth, through root hairs and at epidermal conjunctions [8]. In addition, plant exudates given off through these wounds provide a nutrient source for the colonizing bacteria and thus create favorable conditions. This model of endophytic root colonization was confirmed by several microscopic studies for a number of plants [9]–[11], including poplar [12], [13]. Alternatively, endophytic bacteria can use vector organisms (e.g. arbuscular mycorrhizae and insects) to gain entrance to the apoplastic spaces to colonize the host plant [14]–[16]. Although likely to occur for many plant species, the involvement of specific vector organisms for endophytic colonization has not been demonstrated to play a role in poplar.

After entering the plant, endophytic bacteria must establish themselves. Once established, they can enhance plant health and/or growth by producing plant growth regulating compounds such as indole acetic acid (IAA), acetoin (3-hydroxy-2-butanone), 2,3-butanediol and cytokinins, or metabolize compounds like phenyl acetic acid (PAA), gamma-aminobutyrate (GABA) or the stress ethylene precursor 1-aminocyclopropane-1-carboxylic acid (ACC) [17]–[23]. Endophytic bacteria can also protect the plant from fungal, microbial or insect infections by producing chitinases [24], mannitol dehydrogenase [25], volatile organic compounds and other molecules with antimicrobial activity [26], and by inducing ultrastructural modifications in plant tissues that hinder their penetration by plant pathogens [27].

*Enterobacter* sp. 638, which was isolated from the stem of a 10-year-old hybrid poplar (*Populus trichocarpa×P. deltoids* cv. H11-11) [12], belongs to the family *Enterobacteriacea* whose endophytic members were identified in several plants species including cucumber [28]–[30], grapevine [31], maize [30], [32] and potato [33], [34]. *Enterobacter* sp. 638 is able to increase by up to 40% the growth of several species of poplar, including the *Populus deltoides*×*P. nigra* cultivars DN-34 [12] and OP367 (unpublished data). *Enterobacter* sp. 638 was also found to provide systemic drought resistance to poplar (S. Taghavi and L. Newman, unpublished). In this study we describe the analysis of the *Enterobacter* sp. 638 genome sequence and use metabolite analysis to confirm the production of phytohormones and antimicrobial compounds. Using quantitative RT-PCR we confirmed the dependence of the production of acetoin and 2,3-butanediol on the presence of sucrose, a major plant sugar, in the growth medium.

Exploitation of the *Enterobacter* sp. 638 genome sequence presents a major path forward to identify via a systems biology approach the key functions in plant growth promotion, plant protection and more generally to validate the model describing endophytic colonization, establishment and interaction with the host plant. These findings can be further translated into comprehensive strategies to increase plant establishment and biomass production, which can be used to improve sustainable agriculture, bioenergy feedstock production on marginal lands, or fight desertification of arid areas.

## Results

### Genome structure and general features

**Figure 1:**
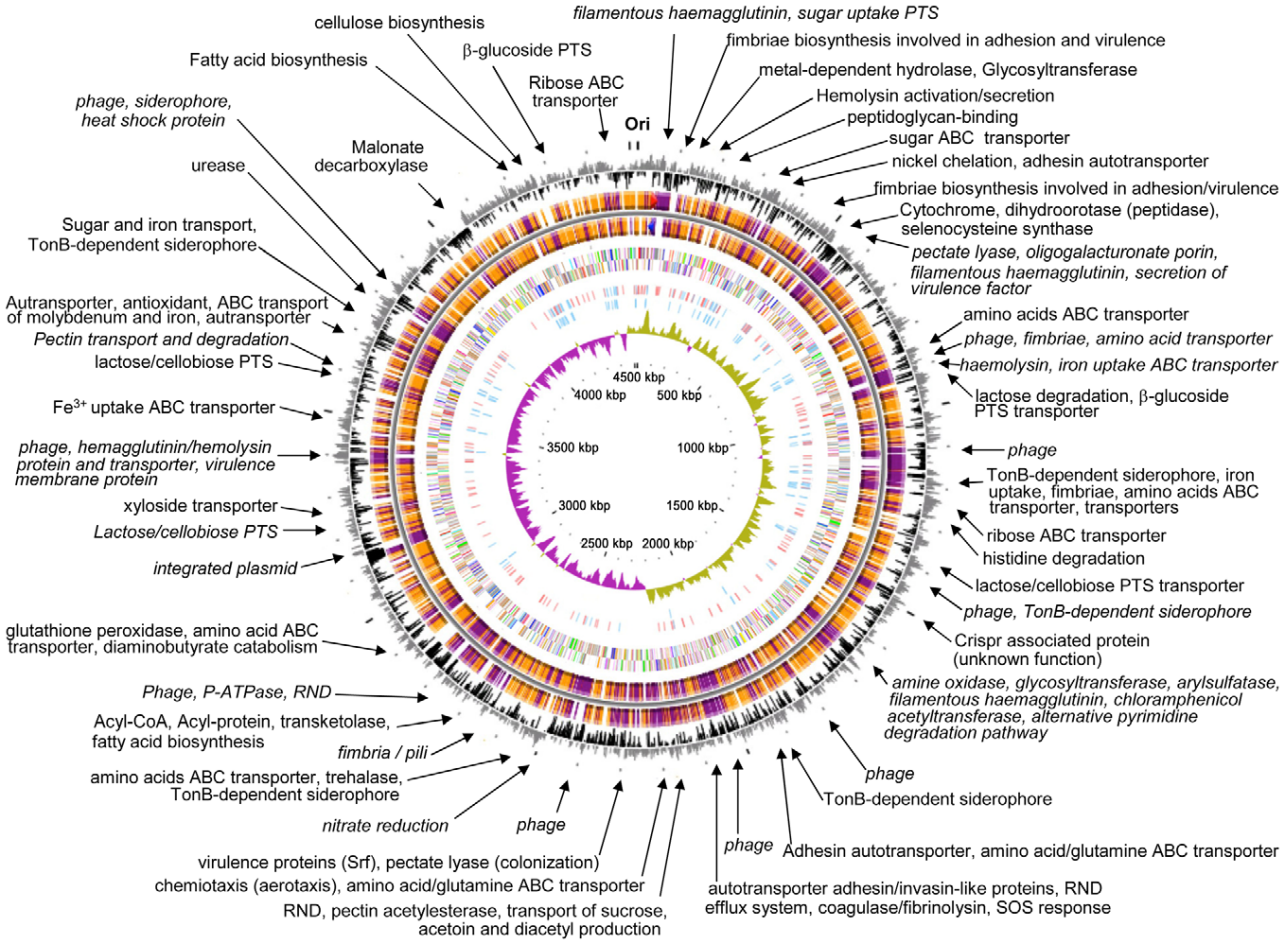
Circular representation of the *Enterobacter* sp. 638 chromosome. Circles displayed (from the outside): the GC percent deviation (GC window - mean GC) in a 1000-bp window, predicted CDSs transcribed in the clockwise direction, predicted CDSs transcribed in the counterclockwise direction, CDS in clockwise and counterclockwise direction colored according to their COG classes, the position of all the palindromic repeats, the position of the 100 palindromic repeats (CCCTCTCCCXX(X)GGGAGAGGG), GC skew (G+C/G−C) in a 1000-bp window, and coordinates in kilo bases pair. Syntenic regions compared with *E. coli* K12 are shown with genes displayed in orange, while genes displayed in purple correspond to non syntenic region. Arrows indicate to putative functions of genes located in region that are not in synteny with *E. coli* K12 (for further detail on gene content for each regions see Table 1). A syntenic region is defined by a minimum of three consecutive genes that are present in *E. coli* K12 genome sequence, and that show a similar genetic organization.

The genome of the gamma-proteobacterium *Enterobacter* sp. 638, a member of the *Enterobacteriaceae*, is comprised of a single circular chromosome of 4,518,712 bp with an overall G+C content of 52.98%, and of a 157,749 bp plasmid pENT638-1 having an overall G+C content of 50.57% (Table 1). The chromosome of *Enterobacter* sp. 638 displays a clear GC skew transition, which corresponds with its replication origin (*oriC*) and terminus (Figure 1). Similar to *E. coli* K12, the *oriC* site contains a perfect DnaA-binding box (TTATCCACA) [35], which is located 31,985 bp upstream of the *dnaA* ATG start codon (at coordinate 4,487,245 bp).

**Table 1:** General features of the *Enterobacter* sp. 638 genome.

General traits	Chromosome	Plasmid
size (bp)	4,518,712	157,749
G+C content	52.98	50.57
ORF numbers	4247	149
Assigned function (including putative)	3457	104
Amino acid biosynthesis	174	2
Purines, pyrimidines, nucleosides, and nucleotides	93	0
Fatty acid and phospholipid metabolism	71	0
Biosynthesis of cofactors, prosthetic groups, and carriers	195	2
Central intermediary metabolism	218	2
Energy metabolism	553	2
Transport and binding proteins	631	3
ABC family	293	2
MFS family	79	2
PTS family	41	0
RND family	14	0
Amino acids, peptides and amines	118	0
Anions	20	0
Carbohydrates, organic alcohols, and acids	106	1
Cations and iron carrying compounds	109	1
Nucleosides, purines and pyrimidines	9	0
Porins	18	0
Unknown substrate or drugs	2	0
DNA metabolism	152	4
Transcription	281	4
Protein synthesis	177	0
Protein fate	188	1
Regulatory functions	515	6
Cell envelope	279	3
Cellular processes	457	6
Biological processes	276	0
RHS	2	0
Plasmid functions	7	42
putative integrated plasmid	1	0
toxin/anti-toxin systems	3	7
Prophage functions	302	0
Phage regions	8	0
integrases	18	2
Complete IS elements	6	2
Unknown function	791	45
No homology to any previously reported sequences	121	26

**Figure 2:**
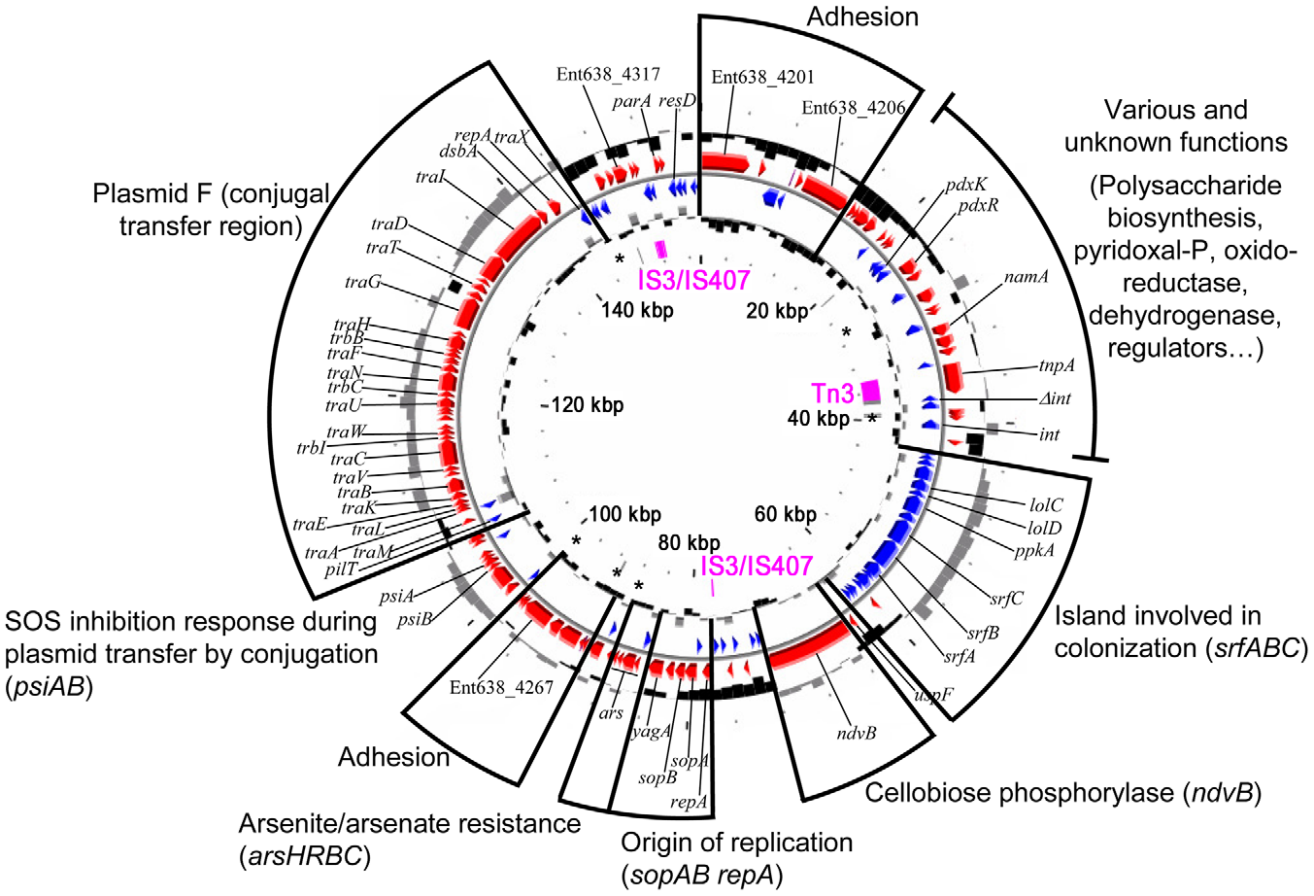
Circular representation of the *Enterobacter* sp. 638 plasmid pENT638-1. Circles displayed from the outside): subdivision of pENT-01 group of function, gene annotation, the GC percent deviation (GC window - mean GC) in a 1000-bp window, predicted CDSs (red) transcribed in the clockwise direction, predicted CDSs (blue) transcribed in the counterclockwise direction, GC skew (G+C/G−C) in a 1000-bp window, transposable elements from IS elements (pink) and pseudogenes (grey). Toxin/anti T toxin (TA) systems are shown with an asterisk (*).

The pENT638-1 plasmid displays, based on GC content, at least four distinct regions (Figure 2). The plasmid is clearly composed of an ancestral backbone, which is common to F-family plasmids [36] and contains the plasmid's basic functions for transfer and replication, and of regions that were likely acquired via horizontal gene transfer. These regions display a codon usage matrix different from the rest of the *Enterobacteriaceae*, have no synteny to sequenced chromosomes or plasmids from closely related strains, and interestingly encode genes related to plant adhesion and colonization. The stable maintenance of pENT638-1 and these specific regions, which presumably play an important role in the successful interactions between *Enterobacter* sp. 638 and its plant host, is assured by the presence of six toxin/anti-toxin (TA) systems, five *relBE* and one *parED*. In contrast, the chromosome of *Enterobacter* sp. 638 encodes only four couples of toxin/anti-toxin systems (*relBE*-like: Ent638_0434-0435; *yeeU-ypjF*-like: Ent638_0476-0477; *hipAB*-like: Ent638_2033-2034; and *chpAR*-like: Ent638_2066-2067). This low number is representative for host-associated organisms [37].

The *Enterobacter* sp. 638 genome encodes 4396 putative coding sequences (CDS), with 4247 CDS encoded on the chromosome representing a coding density of 87.9%. Plasmid pENT638-1 encodes 149 putative CDS having a coding density of 80.4%. After their manual annotation, 3561 CDS (81%) could be assigned to putative biological functions, while 836 CDS (19%) were annotated as hypothetical proteins of unknown function. For the CDS with unassigned functions, conserved hypothetical proteins are represented by 689 CDS (15.7%), while 147 CDS (3.3%) had no homology to any previously reported sequence. Using the COGnitor module from the MaGe system, 3597 CDS (81.8%) could be assigned to one or more COG functional classes (see Figure S1). The repartition of *Enterobacter* sp. 638 CDS among the different COG classes is very similar to what is observed for *E. coli* K12 [38]. The three most abundant classes are amino acid (E), carbohydrate (G) and inorganic iron (P) transport and metabolism and represent more than 37% of all CDS, pointing to the symbiotic life styles of *Enterobacter* sp. 638 and *E. coli* K12 that require efficient uptake of host-provided nutrients. Seven sets of 5S, 16S, 23S rRNA genes and one additional 5S rRNA gene were found. A total of 83 tRNA genes with specificities for all 20 amino acids, and a single tRNA for selenocysteine were identified. The genome of *Enterobacter* sp. 638 encodes 8 Sigma factors: *fliA* (Ent638_2509; Sigma 28), three *rpoE*–like Sigma 24 (Ent638_3060, Ent638_3117 and Ent638_3389), *rpoS* (Ent638_3212, Sigma 38), *rpoD* (Ent638_3473, Sigma 70), *rpoN* (Ent638_3638, Sigma 54) and *rpoH* (Ent638_3865, Sigma 32). *Enterobacter* sp. 638 has an active *dam* methylase involved in the adenine methylation at GATC sites, as was confirmed by *Mbo*I and *Sau3A*I endonuclease digestion of the DNA, the first enzyme being unable to digest the methylated *Enterobacter* sp. 638 DNA. On the genome of *Enterobacter* sp. 638 we found one hundred palindromic repeats (CCCTCTCCCXX(X)GGGAGAGGG) unevenly distributed over the chromosome (see Figure S2). These hairpin loop forming repeats (with XX(X) mainly being TGT/ACA or AC/TG) are often located in duplicate or triplicate at the 3′ end of genes and presumably play a role in transcription termination.

Six Insertion Sequence (IS) elements were found on the *Enterobacter* sp. 638 chromosome: two from the IS*3*/IS*51* family (one composed of three ORFs with a frameshift (Ent638_0739, Ent638_0740, Ent638_0741) and one composed of a single ORF (Ent638_0060)), one IS element from the IS*110* family (Ent638_1530), and three IS elements from the IS*481* family (Ent638_2980, Ent638_3160 and Ent638_3288). Several IS elements were delimitating putative genomic islands (see section below). Plasmid pENT638-1 possesses two complete IS elements, one from the Tn*3* family composed of one ORF (Ent638_4224) and one from the IS*3*/IS*407* family composed of two ORFs (Ent638_4320 and Ent638_4321), as well as two truncated transposases from the latter family. The complete IS and the truncated transposase from the IS*3*/IS*407* families are flanking a large region encoding genes involved in plasmid maintenance and replication (*sopAB*, *repA*) and genes involved in plasmid transfer by conjugation (*tra*). This 75 kb region can be considered as the pENT638-1 backbone. A similar organization was found on the *Yersinia pestis* plasmid pG8786 (pFRA plasmid family). This family of plasmids is generally involved in host interaction and virulence [39], and based on the numerous genes related to plant adhesion and colonization, a similar role is predicted for plasmid pENT638-1.

The specific adaptation of *Enterobacter* sp. 638 to its plant host was scrutinized through genome comparison with other plant associated microbes and the gastrointestinal bacterium *E. coli* K12 (MG1655). This strain, chosen as a reference organism because it represents the best annotated bacterial genome to date, shared (criteria 80% of identity on 80% of the protein length) 2938 syntenic CDS (69.2% of their genome) with *Enterobacter* sp. 638. When comparing the genome of *Enterobacter* sp. 638 with those of other closely related strains, we found that *Enterobacter cancerogenus* ATCC 35316 showed the closest homology with 80.4% of the CDS in synteny, followed by *Klebsiella pneumoniae* 342 and MGH 78578 (both with 74% of the CDS in synteny), *Citrobacter koseri* ATCC BAA-895 (73%), and the *Escherichia coli* species (between 63 to 73%) (Table S2). The syntenic regions are grouped in 304 syntons with an average number of 10.5 CDS per synton. Fifty-six regions were identified on the *Enterobacter* sp. 638 genome, which were not in synteny with the genomes of closely related bacteria. Among them, eighteen regions met the criteria for putative genomic islands (highlighted in grey in Table S3). These genomic islands carry genes encoding proteins involved in sugar transport (PTS system), adhesion, pectate utilization, iron uptake through siderophore receptors, nitrate reduction, pilus biosynthesis, as well as many others transporters and regulators. Region 47 is illustrative for the acquisition of a genomic island containing genes involved in adaptation to an endophytic lifestyle. This region encodes a putative pectate transporter and degradation pathway, which may allow strain 638 to grow on pectate, an important plant synthesized compound as a carbon source. This genomic island is flanked by an integrase gene and inserted into a tRNA-Gly site.

Eight prophages and one putative integrated plasmid were found on the chromosome. A total of 302 phage proteins, including 18 putative integrases, were identified. In addition, the *Enterobacter* sp. 638 chromosome contains a region with Clustered Regularly Interspaced Short Palindromic Repeats (CRISPR) located next to six genes (Ent638_1401-1406) encoding CRISPR-associated sequences (Cas) [40]. CRISPR are likely to provide acquired resistance against bacteriophages. Six of the eight prophages are flanked by regions that lack synteny with the corresponding regions in closely related bacteria such as *E. coli* K12, O157-H7 and UTI89, *Klebsiella pneumoniae* MGH 78578 or *Citrobacter koseri* BAA-895, and that may have been acquired through phage transduction. These regions contain genes important in bacteria/plant interactions such as amino-acid and iron/siderophore transporters, haemolysin (HCP), and a hemagglutinin protein and transporter (Table S3, Figure 1). Until now, the inter- or extra-cellular mobility of the genomic islands, phages and IS elements was not experimentally demonstrated.

### Survival in the plant rhizosphere: overview of *Enterobacter* sp. 638 metabolic capabilities

**Figure 3:**
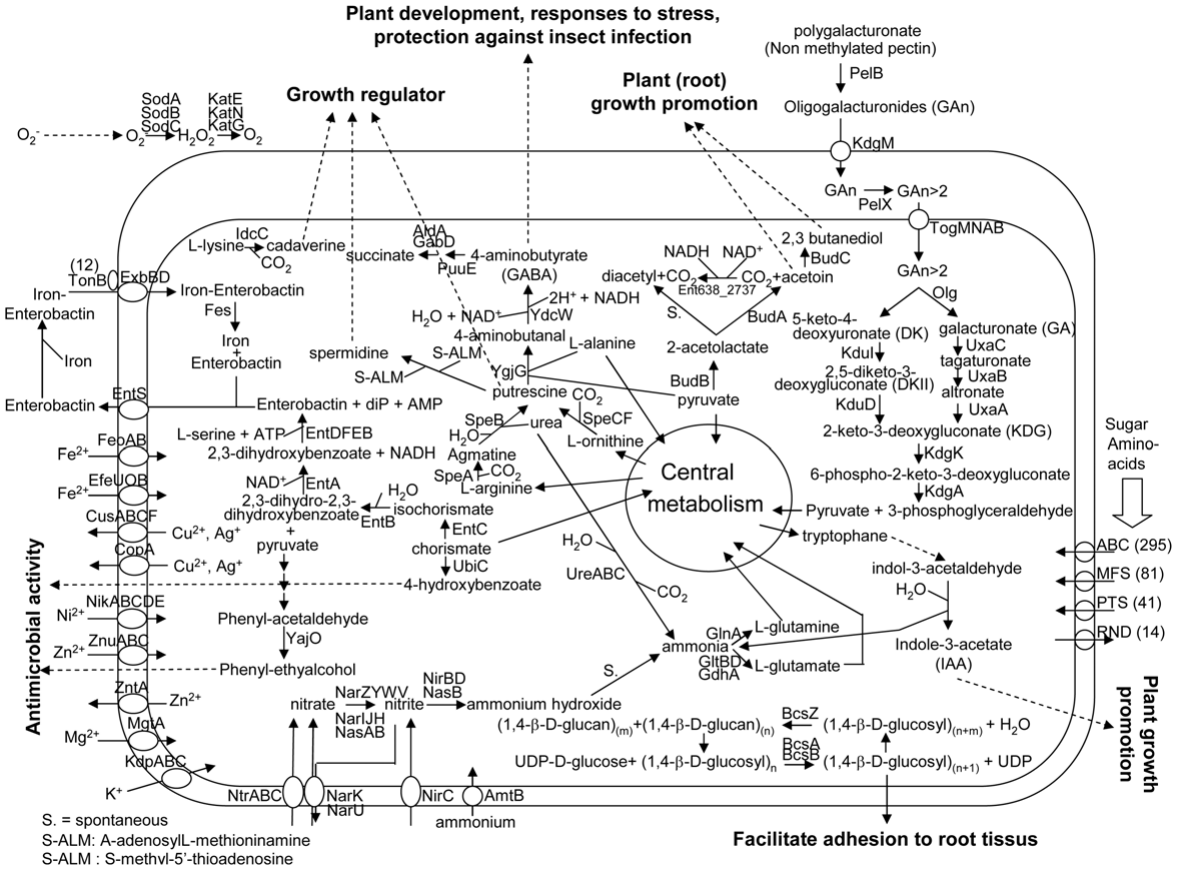
Overview of metabolism and transport in *Enterobacter* sp. 638. Predicted pathways for the interaction between *Enterobacter sp.* 638 and the plant are shown. Export or import of solutes is designated by the direction of the arrow through the transporter. The dash arrows show the putative function of certain molecular biosynthesized by *Enterobacter* sp. 638 in regard to its plant host.

In some plant species, endophytic bacteria are present in the seeds [41]–[43] by means of which they are transferred to the next generation. In general, poplar is propagated by cuttings. Since the number of endophytes in cuttings are very low, as was shown by microscopic studies [44], many species of endophytic bacteria have to survive in the soil prior to colonizing poplar. Based on the genome analysis, *Enterobacter* sp. 638 seems well adapted to survive in the plant rhizosphere because it encodes many transporters involved in carbohydrate, amino-acids and iron uptake, as well as some heavy metal resistance genes. An overview of the metabolic properties and important transport pathways for interactions between *Enterobacter* sp. 638 and its plant host is presented in Figure 3.

#### Carbohydrate metabolism

The *Enterobacter* sp. 638 genome encodes various pathways for central metabolism, including the tricarboxylic acid cycle, the Entner-Doudoroff, the Embden-Meyerhof-Parnas and the pentose-phosphate pathways. The strain is unable to grow autotrophically, but can use a large variety of plant derived compounds as carbon sources: D-mannitol, sucrose, arbutin, salicin, trehalose, D-mannose, L-arabinose, maltose, cellobiose, xylose, gluconate and glucose [12]. *Enterobacter* sp. 638 possesses a lactase (*lacZ*, Ent638_0928), a xylose isomerase (Ent638_0156) and a xylulokinase (Ent638_0157). Lactose utilisation as a sole carbon source is a characteristic of the *Enterobacteriaceae*. *Enterobacter* sp. 638 has the genetic capability to grow on malonate, and its genome contains a cluster of nine genes (*mdcABCDEFGHR*, Ent638_3779-Ent638_3772) involved in malonate decarboxylation to acetate. Various bacteria are able to grow aerobically or anaerobically on malonate as a sole carbon and energy source [45]. A similar group of genes was found on the genome of the endophyte and opportunistic pathogen *K. pneumoniae* 342, but not on the genomes of *E. coli* K12 and the poplar endophyte *Serratia proteamaculans* 568. Malonyl-CoA is the precursor of malonate and is very abundant in plants. The malonate metabolizing genes are important for symbiosis between the bacteria and the plant, as previously shown for *Rhizobium leguminosarium* bv *trifolii* and clover [46].

The diversity of sugar utilization might be related to the diversity of glycoside hydrolases. The *Enterobacter* sp. 638 genome carries 55 genes coding putative glycoside hydrolases, representing 24 different families (CAZy database, http://www.cazy.org/geno/399742.html). Another endophytic *Enterobacteriaceae* isolated from poplar, *S. proteamaculans* 568, shows a similar diversity of glycoside hydrolases (53 genes representative of 20 different families). By comparison, the genome of *E. coli* K12 encodes 43 glycoside hydrolases and an average of 42 was found on the genome sequence of others *E. coli* strains. In contrast, the human pathogen *Enterobacter sakazakii* possesses 63 glycoside hydrolases (http://www.cazy.org/geno/290339.html). Plant pathogenic bacteria and fungi gain access by actively degrading plant cell wall compounds using glycoside hydrolases including cellulases/endoglucanases (including members of the glycoside hydrolase families GH5, GH9, GH44, GH48 and GH74), lichenases (GH16) and xylanases (GH10, GH11). No glycoside hydrolases representing putative members of endo-, exo-, cellulase and hemicellulase families commonly used to break down plant cell wall polymers were encoded on the *Enterobacter* sp. 638 genome. This observation is consistent with the non phytopathogenic behaviour of *Enterobacter* sp. 638. However, it should be noted that two endo-1,4-D-gluconases (GH8) (*bcsZ*: Ent638_3928, Ent638_3936) were found as part of a bacterial cellulose synthesis locus.

#### Uptake of plant nutrients

Organisms living in endophytic association, like *Enterobacter* sp. 638 and its poplar host, need to share resources. Therefore, it is expected that the genome of *Enterobacter* sp. 638 would codes for a large diversity of transporters that will allow for the uptake of plant-produced nutrients. A total of 631 ORFs encode putative transporter proteins: among them 293 coded for ABC transporters (including one phosphate transporter), 79 coded for transporters from the major facilitator superfamily (MFS), 41 coded for transporters from the phosphotransferase system family (PTS) and 14 encoded transporters from the resistance nodulation and cell division family (RND) (see complete list of putative transporters and there substrates in Table S4). This number of transporters is similar to that found on the genomes of the human pathogen *E. coli* O157:H7 (577 ORFs), and of the plant pathogens *Erwinia carotovora* SCRI104 and *K. pneumoniae* MGH78578, with 631 and 521 ORFs respectively. It is higher than for *E. coli* K12, which contains 534 ORFs encoding putative transporters. The higher number for *Enterobacter* sp. 638 compared to the *E. coli* strains results from an overrepresentation of ABC transporters (210 in *E. coli* K12, 239 in *E. coli* O157:H7) and of MFS transporters (70 in *E. coli* K12, 76 in *E. coli* O157:H7) (Table S4). This observation is consistent with the plant associated life style of *Enterobacter* sp. 638, which requires efficient uptake of plant synthesized nutrients, including those released into the rhizosphere. The *Enterobacter* sp. 638 genome encodes 41 PTS transporters. The high number of PTS genes found in the *Enterobacteriales* compared to *Pseudomonadales* and *Xanthomonadales* is well known [47]. Phylogenetic analysis was used to assign substrate specificity to the *Enterobacter* sp. 638 PTS transporters: 7 belonged to the α-glucosides (for uptake of glucose, N-acetylglucosamine, maltose, glucosamine and α-glucosides), 7 to the β-glucosides (for uptake of sucrose, trehalose, N-acetylmuramic acid and β-glucosides), 2 were fructose PTS transporters (for uptake of fructose, mannitol, mannose and 2-O-α-mannosyl D-glycerate) and 6 were lactose PTS transporters (for uptake of lactose, cellobiose and aromatic β-glucosides).

#### Resistance to heavy metals

The *Enterobacter* sp. 638 genome carries genes putatively involved in copper resistance, including a P-type ATPase CopA (Ent638_0962) whose expression is regulated by CueR (Ent638_09630), the copper efflux operon *cusABCF* (Ent638_1157-1154), the multiple copper oxidase CueO (Ent638_0671), and an operon coding for the putative CopC and CopD copper resistance proteins (Ent638_2411-12). Interestingly, the strain failed to grow on 284 glucose minimal medium in the presence of 100 µM Cu(NO_3_)_2_. The *Enterobacter* sp. 638 genome also encodes an arsenic/arsenate resistance cluster that was found next to the origin of replication of plasmid pENT638-1 (*arsHRBC*, Ent638_4254-Ent638_4257), and strain 638 was found to grow successfully on 284 glucose minimal medium in the presence of 200 µM arsenate (as Na_2_HAsO_4_). The presence of arsenate and putative copper resistance genes is not unexpected, as *Enterobacter* sp. 638 was isolated from poplar growing in the area which was impacted by emissions from the ASARCO smelter in Tacoma, WA, a copper smelter that during operations from 1905 through 1982 was considered to be one of the largest arsenic emission sources in the USA. Other heavy metal resistance genes located on the chromosome include a putative chromate reductase (YieF or ChrR, Ent638_4144) and a P-type efflux ATPase ZntA (Ent638_3873) involved in zinc/cadmium/cobalt resistance. Strain 638 was able to grow on 284 glucose minimal medium in the presence of 500 µM ZnSO_4_, 500 µM CdCl_2_, 100 µM CoCl_2_, and 50 µM NiCl_2_. Although it could be argued that these genes are also present in other *E. coli* species, their presence may be enough to provide a selective advantage over other bacteria to survive in the rhizosphere, especially when these metals are present.

Heavy metals are also important cofactors, and the *Enterobacter* sp. 638 genome encodes several genes involved in heavy metal uptake and efflux. Genes were found for ABC transporters involved in zinc (*znuACB*, Ent638_2426-2428) and nickel (*nikABCDE*, Ent638_1834-Ent638_1838) uptake. Nickel is an essential cofactor for urease [48], and unlike *E. coli* K12 and *S. proteamaculans* 568, *Enterobacter* sp. 638 is able to convert urea into ammonia (*ureABC*, Ent638_3464-Ent638_3466).

#### Oxidative stress, counteracting the plant's defense mechanism

Plants use a variety of defense mechanisms against bacterial, viral and fungal infections, including the production of reactive oxygen species (ROS) (superoxide, hydroperoxyl radical, hydrogen peroxide, hydroxyl radical species), nitric oxide and phytoalexins [49], [50]. Prior to root colonization, strain 638 has to survive in an oxidative rhizosphere environment. The *Enterobacter* sp. 638 chromosome encodes three superoxide dismutases: SodA, a Mn superoxide dismutase (Ent638_4063); SodB a Fe superoxide dismutase (Ent638_1191); and SodC, a Cu/Zn superoxide dismutase (Ent638_1801). It also contains three catalases, KatE (Ent638_1712), KatN (Ent638_3129) and KatG (Ent638_4032), three hydroperoxide reductases, *ahpC* (Ent638_0872 and Ent638_1145) and *ahpF* (Ent638_1146), two additional hydroperoxide reductases (a putative *ahpC* Ent638_3391 and Ent638_0498 having an AhpD domain), a chloroperoxidase (Ent638_1149), and two thiol peroxidases (Ent638_2151 and Ent638_2976). We also identified a putative organic peroxide resistance protein, *ohr* (Ent638_0518) located next to its organic peroxide sensor/regulator (*ohrR*, Ent638_0519). *Enterobacter* sp. 638 seems able to detoxify free radical nitric oxide by the presence of a flavohemoprotein nitric oxide dioxygenase (Ent638_3037) and an anaerobic nitrate reduction operon (*norRVW*, Ent638_3181-3183). The expression of the oxidative stress response systems is controlled via complex regulatory networks [51]. A key regulator is the hydrogen-peroxide sensor OxyR (Ent638_4025), which activates the expression of a regulon of hydrogen peroxide-inducible genes such as *katG*, *gor* (glutathione reductase, Ent638_3913), *ahpC*, *ahpF*, *oxyS* (a regulatory RNA, Ent638_misc_RNA_29), *dpsA* (a DNA protection during starvation protein, Ent638_1299), *fur* (a DNA-binding transcriptional dual regulator of siderophore biosynthesis and transport, Ent638_1198) and *grxA* (glutaredoxin, Ent638_1364), all of which are present in *Enterobacter* sp. 638. Three glutathione S-transferase (GST) genes (Ent638_0139, Ent638_0268 and Ent638_1329), a glutathione ABC transporter (GsiABCD, Ent638_1323-1326), two glutathione peroxidase (Ent638_1732 and Ent638_2699), a gamma-glutamate-cysteine ligase (GshA, Ent638_3168), glutathione synthetase (GshB, Ent638_3351) and gamma-glutamyltranspeptidase (GGT, Ent638_3850) were found on the genome of *Enterobacter* sp. 638. This number is lower than what is found on *E. coli* K12 (4 GST genes) and *K. pneumoniae* 342 (12 GST genes) [52]. An AcrAB (Ent638_0943-0944) locus, belonging to RND family of transporters and required for the export of apple tree pytoalexins by *Erwinia amylovora* and the successful colonization of the host plant [53], was also identified on the *Enterobacter* sp. 638 genome.

### Step 1: Moving toward the poplar roots: motility/chemotaxis

As described in the introduction, endophytic colonization of a plant host can be divided into four step process [54], [55].

Motility is an important characteristic for endophytes. Although endophytic bacteria can follow water fluxes for passive movement, they also need to be able to move inside the plant since endophytes tend to colonize specific plant parts that don't always correspond to the port of entry in the plant. *Enterobacter* sp. 638 is well equipped to actively move towards plant roots, the preferred site of endophytic colonization. Its genome contains three flagellar biosynthesis operons (*flgNMABCDEFGHIJKL*, *flhEAB fimA yraIJ lpfD cheZYBR tap tar csuEDCAB int cheWA motBA flhCD fliYZA fliCDSTEFGHJKLMNOPQR*, Ent638_2445-2541 and *fliEFHIJKLMNOPQR*) which are very similar to those found in *Salmonella enterica* subsp. *Enterica typhi* and *E. coli* K12. However, the *flh* operon of *Enterobacter* sp. 638 contains two insertions of pili biosynthesis genes. One of these regions (*csu*) is flanked by an integrase, pointing to later acquisition. *Enterobacter* sp. 638 also has a larger number of pilus/fimbriae biosynthesis genes (at least 60 genes) than *E. coli* K12 (42 genes). In both bacteria, the pilus/fimbriae biosynthesis genes are grouped in 10 distinct regions. Inside the flagellar biosynthesis gene cluster we also found determinants involved in chemotaxis (*che*).

### Steps 2 and 3: Adhesion and colonization of the roots surface

Root adhesion of plant associated bacteria is believed to occur in two steps: non-specific adhesion, followed by firm anchoring. The end of the roots (apex) and the ramification of roots are known to be the primary target for endophytic invasion of plants. In *Enterobacter* sp. 638, several genes were identified encoding proteins involved in the putative adhesion to the root. Many are located on genomic islands or on plasmid pENT638-1, pointing towards a specific role of this plasmid during this step of plant root colonization. In particular, pENT638-1 contains a 23kb putative genomic island, flanked by an integrase gene and having a GC% of 56.2, which is significantly higher than for the plasmid (50.57%). This genomic island contains a group of ORFs that display strong homology to hypothetical proteins found in *Azotobacter vinelandii* AvOP, as well as a putative *srfABC* operon, which is also present in a horizontally acquired region of *Salmonella* spp [56]. A second copy of the *srfABC* operon (Ent638_2108-Ent638_2110) was found on the *Enterobacter* sp. 638 chromosomes. The *srf* genes were also found in plant and animal colonizing bacteria (*Yersinia sp.*, *Salmonella sp.*, *Pectobacterium atrosepticum*, *Enterobacter sakazakii*) and in the poplar endophyte *S. proteamaculans* 568. The exact function of the *srfABC* operon remains unclear, but it is believed to be involved in host colonization [56].

#### Hemagglutinin

Bacterial colonization relies on a variety of cell surface-associated factors that allow adhesion to the host surface. Filamentous hemagglutinin-like adhesins are important in both plant [57] and animal pathogens [58]. The chromosome of *Enterobacter* sp. 638 encodes two putative hemagglutinin proteins (Ent638_0148, Ent638_3119), and a cluster composed of five genes encoding for the synthesis of a filamentous hemagglutinin (Ent638_0052-0057), which based on sequence homology is very similar to the CdiA protein of *E. coli*, where it is responsible for contact-dependent inhibition of growth [59], [60]. In addition, several genes were found on the chromosome of *Enterobacter* sp. 638 that code for autotransporter proteins with a pectin lyase/pertactin domain (Ent638_1775, Ent638_0318, Ent638_0501), or an adhesion domain (*yadA*, Ent638_1867; Ent638_3408).

The two *yadA* genes (Ent638_1867; and Ent638_4317 on pENT638-1) code for proteins with an autotransporter domain and an invasin/adhesion domain that are normally involved in mammalian cell invasion by *Enteric*/*Yersinia* bacteria. The YadA protein might promote plant colonization/invasion, but could also represent a remnant of an ancient enteric lifestyle. However, it cannot be ruled out that *Enterobacter* sp. 638 is able to invade eukaryotic cells. Several additional genes putatively involved in plant invasion are present on pENT638-1 including proteins with an autotransporter domain (secretion type V) and a virulence/adhesion domain: hemagglutinin (Ent638_4267), pertactin (Ent638_4201 and Ent638_4206) (Figure 2). The hemagglutinin gene on pENT638-1 (Ent638_4267) is surrounded by two RelB/E toxin/anti-toxin systems. It is hypothesized that the Ent638_4267 hemagglutinin must play an important role in root adhesion to have been stabilized in this way on pENT638-1. Adjacent to the hemagglutinin gene we found two genes (Ent638_4265-4266) that code for proteins containing a tetratricopeptide (TPR-2) repeat domain, putatively involved in protein-protein interaction and the correct assembly of the adhesion apparatus.

#### Type I and IV pili

The chaperon/usher pathway is a delivery system for type-I pili, which are synthesized in the periplasm, across the Gram-negative outer membrane [61]. Six putative usher proteins were found on the *Enterobacter* sp. 638 genome (Ent638_0084, Ent638_0403, Ent638_0990, Ent638_1071, Ent638_2450, and Ent638_2459). This number is very similar to the average of eight usher units found on the genomes of enteric bacteria of the genera *Escherichia*, *Salmonella*, *Shigella* and *Yersinia* [52], [61]. It is, however, much higher than the average number of usher proteins found in other genera of plant associated bacteria (only one was found on the *Erwinia*, *Agrobacteria*, *Xanthomonas* and *Xylella* genomes, and two on the *Pseudomonas* sp. genomes).

Type I pili are widely distributed in enteric bacteria and are composed of several different protein components, including an adhesin that is part of the short thin fibrillar pilus tip, and FimA, which binds to mannose sugars present on a variety of different host cell surface structures. On the *Enterobacter* sp. 638 chromosome, 56 genes involved in pili/curli/fimbriae biosynthesis were identified, including 6 clusters of type-I pili biosynthesis genes (Ent638_0074-0086, Ent638_0401-0409, Ent638_0987-0994, Ent638_1068-1072, Ent638_2448-2451, Ent638_2458-2462). The last two clusters are flanked and separated by genes involved in chemiotaxis and motility (flagellar biosynthesis - see section motility), and are possibly involved in biofilm formation on abiotic surfaces [62]. This region (Ent638_2445-2541) represents an example of clustering genes putatively involved in different aspects of plant root colonization (chemiotaxis, motility, and adhesion).

#### Type IV pili

Type IV pili are displayed by a wide variety of Gram-negative bacteria. They have a distinctive method of assembly involving proteolytic processing and N-methylation of the pilin precursor. Although components of type IV pili can be involved in natural DNA uptake (transformation) in *Neisseria*, they are primarily involved in adhesion to surfaces and are among the few factors known to affect endophytic colonization [63]. The establishment of microcolonies on roots and fungal mycelium, and the systemic spreading in rice are also mediated by type VI pili [63]. On the *Enterobacter* sp. 638 genome two clusters of type-IV pili biosynthesis genes were identified (Ent638_0650-0652, and Ent638_3266-3268) as well as a cluster of putative uncharacterized pilus biosynthesis genes (Ent638_3804 and Ent638_3808) that are possibly involved in DNA uptake.

#### Curli fibers

Curli fibers are involved in adhesion to surfaces, cell aggregation and biofilm formation, and also mediate host cell adhesion and invasion. The structure and biogenesis of curli are unique among the bacterial fibers that have been described to date. Structurally and biochemically, curli belongs to a growing class of fibers known as amyloids. On the genome of *Enterobacter* sp. 638, one cluster for curli biosynthesis (Ent638_1553-1559) was identified.

#### Cellulose biosynthesis

The genome of *Enterobacter* sp. 638 does not encode proteins involved in cellulose degradation, which is consistent with its non pathogenic behaviour. However, an operon responsible for bacterial cellulose biosynthesis was identified (Ent638_3927-3940). Bacterial cellulose production may enhance the adhesion of endophytic bacteria to root tissue [64].

#### Virulence

Microsopic studies showed that *Enterobacter* sp. 638 colonizes the root xylem between the lumen of the lenticels [12]. Although *Enterobacter* sp. 638 was not found to act as an opportunistic pathogen in plant colonization studies, its genome was found to code for several proteins putatively involved in virulence. We found one gene (*yqfA*, Ent638_3317) coding for an inner membrane hemolysin (family III), a partial CDS (Ent638_0251) containing a putative hemolysin domain, and three *hcp* genes putatively coding for virulence factors (Ent638_0829, Ent638_2912 and Ent638_3004). The Hcp effector, a hexameric protein forming a ring, was found in pulmonary secretion of cystic fibrosis patients [65]. In *Pseudomonas aeruginosa* species isolated from cystic fibrosis patients, the Hcp virulence factor is exported through a protein secretion apparatus that was absent from the *Enterobacter* sp. 638 genome. Other putative virulence factors include *pagC* (Ent638_3136) and *msgA* (Ent638_1656), which are required for virulence and survival within macrophages, and two putative *virK* genes (Ent638_1394 and Ent638_2409), whose product is required for the expression and correct membrane localization of VirG (Ent638_3560) on the bacterial cell surface [66]. However, genes encoding for a type III secretion system, which is a prerequisite for an active virulent life style typical for pathogens such as *Erwinia* sp. and *Pseudomonas syringae*, were absent from the *Enterobacter* sp. 638 genome.

### Step 4: Invasion of the root and *in planta* establishment via active colonization

Endophytic bacteria can enter the plant root at sites of tissues damage. This seems to be a major mode of entry for *Enterbacter* sp. 638 as it genome doesn't encode endo/exo-cellulases or hemicellulases that would allow endophytic colonization via a process involving active breakdown of plant cell walls.

#### Pectin/pectate degradation

Pectin is located throughout the primary cell walls and in the middle lamella between plant cells helping bind cells together. Although *Enterobacter* sp. 638 is not able to grow on pectin (poly(1,4-alpha-D-galacturonate)) as a sole carbon source, its genome contains a genomic island encoding the genes involved in the degradation of pectate, the demethylated backbone of pectin and a constituent of the plant cell wall. The ability of *Enterobacter* sp. 638 to degrade pectate could play a role in colonizing the interspatial region between plant cells. A secreted pectate lyase, PelB, involved in the cleavage of pectate into oligosaccharides with 4-deoxy-alpha-D-galact-4-enuronosyl groups at their non-reducing ends, was found next to an oligogalacturonate-specific porin, KdgM, involved in the uptake of oligogalacturonides into the periplasm. A periplasmic pectinase, PelX, encoded by a different region of the genome, is involved in periplasmic degradation of oligogalacturonide.

A carbohydrate uptake ABC transporter, TogMNAB, involved in the translocation of oligogalacturonide across the inner membrane and several additional proteins, Ogl, KduI and KduD, involved in the degradation of oligogalacturonide into 2-dehydro-3-deoxy-D-gluconate are present on a second genomic region. It also contained KdgK and KdgA that further degrade 2-dehydro-3-deoxy-D-gluconate into pyruvate and 3-phosphoglyceraldehyde, both compounds of the general cellular metabolism. This region, which is flanked by a transposase of the IS*481* family, might have been acquired via horizontal gene transfer. The proteins UxaA, UxaB, and UxaC that provide an alternative pathway to degrade galacturonate into 2-dehydro-3-deoxy-D-gluconate, are also found on the *Enterobacter* sp. 638 chromosome. The degradation of pectate has to be well regulated in order to avoid a pathogenic effect.

Plasmid pENT638-1 carries two neighbouring genes (Ent638_4201, Ent638_4206) encoding for autrotransporter proteins with a pectin lyase domain. It is unclear if these proteins are involved in the adhesion of *Enterobacter* sp. 638 to poplar roots or if these proteins are part of a colonization mechanism that involves the export of enzymes able to lyse the root cell walls. Between these two genes we found two component transcriptional regulators, suggesting a tight regulation, as well as two additional genes involved in capsular polysaccharide biosynthesis (Ent638_4207) and encoding for a glycosyl transferase (Ent638_4208). Cell surface lipopolysaccharides (LPS) have been hypothesized of being involved in host specificity, and the proximity of these genes suggests a collaborative role in plant invasion by *Enterobacter* sp. 638.

#### The pENT638-1 plasmid cellobiose phosphorylase

On plasmid pENT638-1, the *ndvB* gene (8532 bp), located next to the plasmid's origin of replication, encodes a protein involved in the production of β-(1->2)–glucan. The membrane bound NdvB protein catalyzes three enzymatic activities: the initiation of protein glucosylation, elongation, and *in situ* cyclization of β-(1->2)–glucan, which is then released into the periplasm [67]. It has been reported that cyclic β-(1->2)–glucan is involved in the attachment of *Agrobacterium tumefaciens* to plant cells [68]. In *Rhizobium meliloti*, mutations of the *ndvB* gene that reduced the amounts of periplasmic β-(1->2)-glucan, resulted in altered phenotypes related to phage and antibiotic sensitivity, motility, and growth in low osmolarity media. Bacteroids produced by two of the downstream mutants were morphologically abnormal, indicating that *ndvB* is involved not only in invasion but also in bacteroid development [69]. The *ndvB* gene is not present in *E. coli* K12, but was identified in two other poplar endophytes, *S. proteamaculans* 568 and *Pseudomonas putida* W619.

### Synergistic interactions with the host plant: plant growth promotion

#### Nitrogen fixation and metabolism

Unlike *rhizobium* and other nitrogen fixing endophytic bacteria, including the poplar endophyte *Rhizobium tropici* [70], *Enterobacter* sp. 638 is unable to fix nitrogen and lacks the required *nif* genes. However, it contains the genes required for dissimilatory and assimilatory nitrate reduction pathways. The nitrate transport and nitrate/nitrite reduction genes are present within two operons (*narIJHGKXL* and *nasAB ntrCBA nasR*, Ent638_2312-Ent638_2326) separated by an integrase and a putative adhesion/invasion gene. Others regions involved in nitrite transport and reduction (*nirBDC*, Ent638_3793-3795), nitrate transport and reduction (*narUZYWV*, Ent638_2061-Ent638_2065), and an ammonium uptake transporter (*amtB*, Ent638_0919) and its regulator (Ent638_0918), as well as the nitrate/nitrite sensor protein (*narQ*, Ent638_2964) were also found on its chromosome.

#### Siderophores

Bacteria have developed several distinct mechanisms to compete for iron, an element whose availability often limits microbial growth. These include specific iron uptake transporters, the secretion of large numbers of diverse siderophores, and the synthesis of siderophore receptors to utilize heterologously produced siderophores from other microorganisms. Siderophores are known to have an antagonistic effect by depriving iron from other microorganisms (for review, see [71]). The presence of an efficient iron uptake system can therefore contribute to protect the host plant against pathogenic infections.

*Enterobacter* sp. 638 has developed an intermediate solution to deal with iron uptake. Its genome contains two ferrous iron uptake systems (FeoAB, EfeUOB) and nine iron ABC transporters. This number is much larger than the four iron ABC transporters found in *E. coli* K12 or the seven operons identified on the *S. proteamaculans* 568 chromosome.

Similarly to *E.coli* K12, *Enterobacter* sp. 638 is able to synthesize the siderophore enterobactin (EntD, EntF, EntC, EntE, EntB and EntA), secrete it (EntS), recover the iron-enterobactin complex using a ferric siderophore uptake system (ExbDB), and extract the iron using an enterobactin esterase (Fes) after internalization of the iron-enterobactin complex. The genes involved in this biosynthesis of enterobactin are grouped with genes encoding two ABC transporters involved in iron uptake (*sitABCD* and *fepCGDB*) in a large cluster of 17 genes (Ent638_1111-1128). Furthermore, *Enterobacter* sp. 638 possesses 12 outer membrane ferric and ferric-related siderophore receptors (TonB dependent), which is almost double of the number found in *E. coli* K12 (that only possesses 7 siderophore receptors). This observation is consistent for a bacterium that needs to compete for iron and can do this via the uptake of heterologous ferric-siderophore complexes.

#### Antimicrobial compounds

Mass spectrometry showed that *Enterobacter* sp. 638 produced 2-phenylethanol in the presence of various carbon sources. 2-Phenylethanol, which has antimicrobial properties, is commonly used in perfumery and gives *Enterobacter* sp. 638 cultures a pleasant floral odor. Two candidate genes (Ent638_1306 and Ent638_1876) encode an enzyme putatively involved in the conversion phenyl-acetaldehyde into 2-phenylethanol. Both genes are located in regions not syntenic with other closely related strains.

A precursor of the important electron carrier ubiquinone, 4-hydroxybenzoate, is also known to have antimicrobial activity. In contrast to *E. coli* K12, which is not able to degrade chorismate into 4-hydroxybenzoate and pyruvate, *Enterobacter* sp. 638 possesses the *ubiC* (Ent638_0243) gene that codes for the putative enzyme able to perform this reaction [72]. In carrot cell cultures and in alfalfa plants, the formation of 4-hydroxybenzoate can be elicited by treatment with funga1 elicitors [73], [74], suggesting a possible role of 4-hydroxybenzoate derivatives as phytoalexins. In addition, it has been reported that 4-hydroxybenzoate stimulates the production of pathogen related proteins in *Nicotianum tabacum*, although to a considerably lower extent than salicylic acid [75].

The *Enterobacter* sp. 638 genome encodes a chloramphenicol acetyltransferase (*cat*, Ent638_1533) which provides resistance to 20 µg/ml chloramphenicol.

#### 1-aminocyclopropane-1-carboxylate deaminase

The 1-aminocyclopropane-1-carboxylate (ACC) deaminase (*acd*), (EC: 3.5.99.7) is absent from the *Enterobacter* 638 genome, which confirms previous studies that the strain is unable to metabolize ACC [12] as a way to moderate the stress ethylene response by its host plant. However, two putative amino acid deaminases were found, but both lack the signature amino-acids E 296 and L 323 (respectively replaced by a T or S and a T) conserved in the active site of ACC deaminases [76].

#### Production of the root growth promoting hormones acetoin, and 2,3-butanediol

Volatile compounds, including a mixture of acetoin and 2,3-butanediol, are emitted by some of the most efficient plant growth promoting rhizobacteria to enhance plant growth [77]. The *Enterobacter* sp. 638 genome carries the gene *poxB* (Ent638_1387) encoding a pyruvate dehydrogenase. While the principal function of PoxB is to convert pyruvate into acetaldehyde, a small fraction of the pyruvate is converted to acetoin, as a by-product of the hydroxyethyl –thiamin diphosphate reaction intermediate [78].

The main pathway for the production of acetoin and 2,3-butanediol by *Enterobacter* sp. 638 is via the *budABC* operon. The acetolactate synthase (*budB*, Ent638_2027) converts pyruvate to acetolactate, which is subsequently converted by the acetoin decarboxylase (*budA*, Ent638_2026) into acetoin. Acetoin is released by the bacteria or subsequently converted into 2,3-butanediol by the acetoin reductase (*budC*, Ent638_2028). Under aerobic condition, acetolactate is spontaneously converted into diacetyl (2,3-butanedione), which in turn can be converted into acetoin by the acetoin dehydrogenase protein (Ent638_2737).

**Figure 4:**
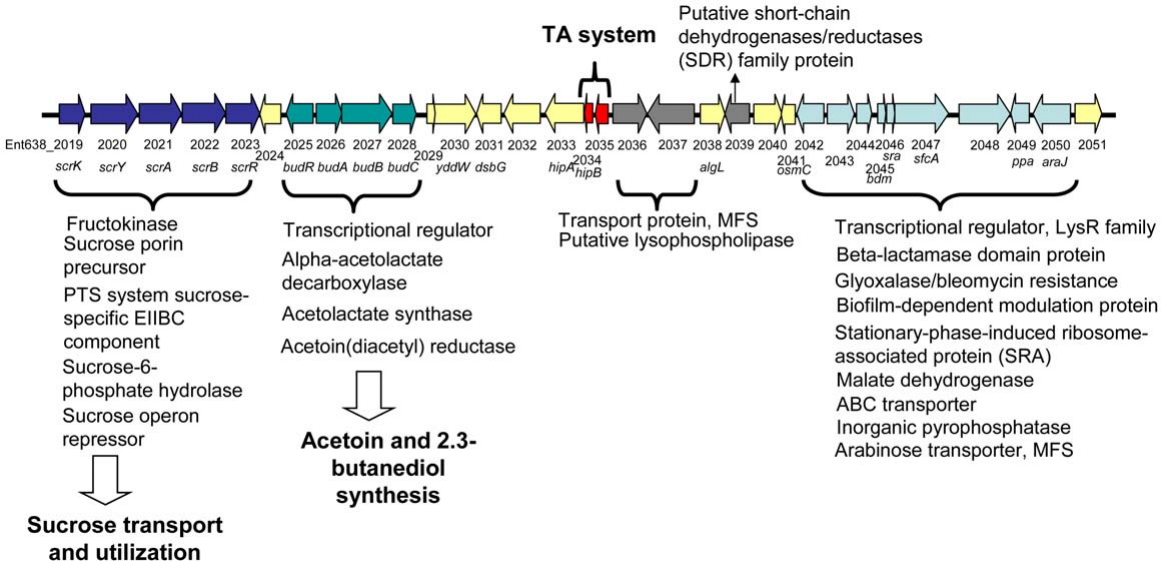
Schematic representation of Genomic Region 29 found on the chromosome of *Enterobacter* sp. 638. Putative open reading frames are indicated by arrows, below which the *Enterobacter* sp. 638 gene number and gene annotation are shown. The genes involved in sucrose transport and utilization, acetoin and 2,3-butanediol synthesis, the toxin-antitoxin (TA system), as well as other putative functions are also indicated.

The *budABC* operon is located on a genomic region (region 29) adjacent to the operon for sucrose uptake and metabolism (see Figure 4 and Table S3). Region 29, which is absent in closely related strains but does not fulfill all criteria to be defined as a genomic island, also encodes several putative transport and regulatory proteins as well as a *hipAB* toxin/anti-toxin system. These features made us hypothesize that region 29 plays an important role in the interaction of *Enterobacter* sp. 638 with its plant host, and that the presence in the growth medium of sucrose, the major photosynthate, might trigger the transcription of the *budABC* operon and the production of the phytohormones acetoin and 2,3-butanediol. Mass spectrometry showed acetoin and 2,3-butanediol synthesis after 12 h for cultures grown on sucrose as sole carbon source (Figure S3), while no synthesis of these compounds was observed when the cultures were grown in the presence of lactate, even after prolonged incubation (24–48h). A similar induction of acetoin and 2,3-butanediol synthesis was observed when the cells were grown in the presence of poplar leaf extracts. The induction of the *budABC* operon in the presence of sucrose was confirmed by quantitative RT-PCR. Very little induction of the *budA* and *budC* genes was observed after 6 hours. However, compared to cells grown on lactate as sole carbon source, cells grown on sucrose showed after 8 hours a 223, 74 and 13 fold induction of the *budA*, *budC* and *scrB* (sucrose-6-phosphate hydrolase, Ent638_2022) genes, respectively (see Table 2).

**Table 2:** Transcription analysis of genes involved in sucrose metabolism, acetoin, and 2,3-butanediol synthesis in *Enterobacter* sp. 638 when grown in the presence of sucrose or lactate as carbon source.

	Induction	
Gene	6 hours	8 hours
*budA*	4.6 (+/−0.1)	223.0 (+/−15.0)
*budC*	2.8 (+/−0.5)	73.5 (+/−5.0)
*scrB*	24.3 (+/−3.2)	13.0 (+/−1.7)
*recA*	1 (+/−0.07)	1 (+/−0.01)

Cultures of *Enterobacter* sp. 638 were grown for either 6 or 8 hours in the presence 0.2% sucrose or lactate as sole carbon source, after which cells were harvested. Their total RNA was isolated, and after removal of 16S and 23S rRNA converted into cDNA and used for Q-PCR analysis. Expression of *recA* (encoding a protein involved in DNA repair) was used as an internal control to normalize gene expression. Results are given in fold induction of cells grown on sucrose as a sole carbon source with respect to the culture on lactate as a sole carbon source, and are mean ± SD (N = 3).

The genome of *Enterobacter* sp. 638 lacks the genes (*acoABCX adh*) involved in the catabolic conversion of acetoin and 2,3-butanediol to central metabolites [79]. Therefore there is no antagonistic effect between the production and the degradation of these phytohormones by *Enterobacter* sp. 638.

#### Production of the plant growth hormone IAA

Endophytic and rhizosphere bacteria also enhance plant growth through the synthesis of the plant auxin indole acetic acid (IAA), which can be synthesized from tryptophan via three alternative pathways [12]: indolepyruvate, tryptamine, or indole-3-acetamide. The production of IAA by *Enterobacter* sp. 638 was experimentally demonstrated and is likely through the production of indolepyruvate as an intermediate molecule by the tryptophane degradation pathway VII (aromatic amino acid aminotransferase, Ent638_1447) [12]. The indolpyruvate decarboxylase IpdC (Ent638_2923) and the putative indole-3-acet-aldehyde dehydrogenases (Ent638_0143) further catalyze IAA synthesis, which is subsequently secreted via an auxin efflux carrier (Ent638_3774). The gene *ipdC*, although not located on a genomic island, is absent from the *E. coli* K12, O157:H7 and UTI89 genomes. Two commonly found alternative pathways for IAA production, via tryptamine or indole-3-acetamide, are either absent or incomplete.

## Discussion

Analysis of the whole genome of the plant growth promoting endophytic bacteria *Enterobacter* sp. 638, which was isolated from the stem of poplar, reveals several features that reflect the dualistic lifestyle of this bacterium: survival in the rhizosphere, which represents a harsh and competitive environment, and endophytic colonization of the relatively protected plant environment. This is represented by the metabolic capabilities of this strain, as we discussed in the results section, and by the architecture of its genome.

The *Enterobacter* sp. 638 genome contains 8 IS elements, which is relatively low compared to other endophytic bacteria such as *K. pneumoniae* 342 and *S. proteamaculans* 568 whose genomes contain 20 [52] and 12 full-length IS elements, respectively, or compared with the 66 IS-like genes found on the genome of *E. coli* K12 [80]. More generally, an endophytic life style may protect the bacteria from the outside environment and therefore requires less genome plasticity. This idea is supported by the example of the clinical isolate *Stenotrophomonas maltophilia* K279a [81], which has with 30 full-length IS elements twice the number of IS elements as found in strain R551-3 [82], an endophytic bacterium to which it is closely related.

The chromosome of *Enterobacter* sp. 638 encodes four toxin/anti-toxin (T/A) systems (*relE/B*, Ent638_0434-0435; *yeeV/U*, Ent638_0476-0477; *hipA/B*, Ent638_2033-2034; and *chpA/R*, Ent638_2066-2067). This low number is representative for host-associated organisms [37]. In contrast, the relative high number of six T/A systems (five *relBE* and one *parED*) found on plasmid pENT638-1 seems to reflect the dualistic life style, rhizosphere and endophytic, of *Enterobacter* sp. 638 [37]. When residing in the rhizosphere the presence of the low copy number (1 to 2 copies per chromosome) 157 kb pENT638-1 plasmid, which besides arsenic resistance does not seem to code for any function that provides a competitive advantage for rhizosphere survival, causes a burden on strain 638. However, the architecture of pENT638-1 seems to reflect an essential role for this plasmid in the endophytic lifestyle of this strain. In addition to the active *parAB* partitioning system, the T/A systems make it virtually impossible to lose this plasmid under non-selective conditions. The various T/A systems are strategically positioned in the proximity of four regions of pENT638-1 that encode putative genes related to plant adhesion and colonization (Figure 2), such as a region coding for a putative hemagglutinin-related autotransporter (Ent638_4267). Based on their different G/C content, these regions were likely acquired via horizontal gene transfer. The T/A systems ensure their stable integration in pENT638-1 under non-selective conditions. In comparison the stable maintenance of plasmid pG8786, the *Y. pestis* pFra virulence-associated plasmid, is enforced by the presence of a single *parAB* partitioning system [39].

*Enterobacter* sp. 638 belongs to the *Enterobacteriaceae*, a genus that contains both beneficial plant associated microorganisms as well as opportunistic pathogens. Other examples of this dualistic life style include *K. pneumoniae*, which both have endophyte (strain 342) and opportunistic pathogen (strain MGH78578) [52] or *S. maltophilia* with the endophytic strain R551-3 and the opportunistic pathogens K279a strain [82]. It is therefore important to assess the pathogenic potential of strain 638. The *Enterobacter* sp. 638 genome annotation and its comparison with other endophytic bacteria and closely related (opportunistic) pathogenic bacteria revealed that strain 638 is lacking a type III secretion system, which is considered a prerequisite for an active virulent life style typical for plant pathogens such as *Erwinia* and *P. syringae*. *Enterobacter* sp. 638 and other well-known endophytic bacteria share many genetic determinants for adhesion and even hemolysin with opportunistic pathogenic bacteria. In fact, plasmid pENT638-1 alone carries four putative genes involved in plant adhesion. These genes are likely essential for colonization and not related to pathogenicity. Therefore, although functions putatively involved in virulence were found, we feel confident to conclude that *Enterobacter* sp. 638 is in overall a beneficial organism and not an opportunistic pathogen. This is supported by the broad plant growth promoting host range of *Enterobacter* sp. 638, which includes tomato, tobacco, poplar, and sunflower.

Our study reveals that the genome of *Enterobacter* sp. 638 carries many genes that make it an interesting candidate for agricultural application, and to improve the growth of biofuel feedstocks such as poplar. The plant growth promoting properties of *Enterobacter* sp. 638 depend on different routes of interactions. *Enterobacter* sp. 638 can indirectly stimulate plant health by protecting its host against bacterial and fungal infections, via the competition for essential nutrients such as iron, and the production of the antimicrobial compounds. For example, production of 2-phenylethanol gives a competitive advantage to *Enterobacter* sp. 638 in the rhizosphere but will also protect its host plant against pathogen infection.

Although *Enterobacter* sp. 638 is able to produce low levels of the phytohormone IAA [12], its major pathway to directly promote plant growth and development seems to rely on the production of acetoin and 2,3-butanediol. Increased levels of these phytohormones stimulate root development and will result in better access to nutrients and water, which will consequently increase growth and establishment of the host plant and better management of available water. In addition, the production of acetoin and 2,3-butanediol by plant growth promoting bacteria was reported to increase systemic disease resistance [83] and drought tolerance [84]. This is consistent with our preliminary observations that *Enterobacter* sp. 638 increases drought tolerance in *Populus deltoides* × *P. nigra* OP-367.

On the metabolome and transcriptome level the production of acetoin and 2,3-butanediol by *Enterobacter* sp. 638 was induced by the presence of sucrose, a sugar abundant in plants. Together with genes coding for sucrose transport and utilization, the *budABC* operon for acetoin and 2,3-butanediol synthesis is located on region 29. Such clustering of genes further supports a relation between sucrose utilization and the inducible synthesis of these phytohormones. It should also be stated that the genome of *Populus trichocarpa* lacks the genes for the biosynthesis of acetoin from pyruvate, but that the gene responsible for the conversion of acetoin into 2,3-butanediol was identified. This points to a remarkable interaction between *Enterobacter* sp. 638 and its poplar host with the endophyte responsible of the production of a phytohormone, and a precursor for another, that poplar is unable to synthesize, and where the production of the plant growth promoting compounds depends on the presence of plant synthesized compounds, such as sucrose, in the growth medium. So far, the *budRABC* operon from *Enterobacter* sp. 638 was found syntenic to the following genomes: *Enterobacter cancerogenus* ATCC 35316, *K. pneumoniae* MGH 78578 and 342, *Enterobacter sakazakii* ATCC BAA-894, *Vibrio alginolyticus* 12G01, and *Vibrio cholerae* N16961, MZO-3, B33, O395, V52, 2740-80, 1587, MAK 757, NCTC 8457, MZO-2 and 623-39.

Other compounds, known to be involved in plant growth promotion, were predicted by genome analysis to be synthesized by *Enterobacter* sp. 638. Since these compounds, which include putrescine, spermidine and cadaverine, are also produced by a large variety of non-endophytic bacteria, their role in plant growth promotion by *Enterobacter* sp. 638 remains to be demonstrated. Another example of a plant growth promoting compound is 4-aminobutyrate (GABA), an important phytohormone and eukaryotic neurotransmitter. GABA is produced by the plant in response to parasite infection and can interact with the brain neurotransmitters of insects. Like *E. coli* K12, *Enterobacter* sp. 638 possesses the genes required for GABA synthesis, but cannot utilize it as a sole carbon or nitrogen source [12]. Annotation reveals that the *Enterobacter* sp. 638 genome lacks the gene encoding for the GABA permease, which makes it questionable if *Enterobacter* sp. 638 can produce and export GABA as a protecting agent.

Overall, the *Enterobacter* sp. 638 genome sequence presents a major tool to identify via a systematic approach the key functions in plant growth promotion, plant protection and more generally to validate the model describing endophytic colonization, establishment and interaction with the host plant. A combination of transcriptomics, proteomics, metabolomics and mutagenesis to study the plant colonization process will be of invaluable help in this respect: it will allow assigning new functions to putative genes and pathways, help to detect new proteins, and confirm the metabolic potential of the strain. As a first step to better understand the endophytic interactions between *Enterobacter* sp. 638 and poplar, a whole genome *Enterobacter* sp. 638 microarray was designed that is currently being validated to study changes in gene expression that occur in strain 638 during the various steps of the endophytic colonization process of poplar. Simultaneously, microarrays studies can provide valuable information on how endophytic colonization affects gene expression in poplar. Changes in gene expression for *Enterobacter* sp. 638 and poplar will provide strong support to identify genes involved in the successful endophytic colonization process, including those putative genes related to plant adhesion and colonization, many of which were coded for by plasmid pENT638-1. Furthermore, gene expression studies combined with metabolite analysis and proteomics will help to better understand mechanisms for the inducible synthesis of phytohormones, signaling compounds and other secondary metabolites that play a role in endophytic colonization and plant growth promotion and development, as was already proven for acetoin and 2,3-butanediol synthesis. Comparative transcriptome and proteome analysis will also provide valuable insights in which other genes and pathways are affected during the endophytic colonization process and the observed stimulation in plant growth and development. Genetic engineering and mutation analysis of *Enterobacter* sp. 638 and poplar should confirm the role of genes and metabolic pathways in successful endophytic colonization and plant growth and development. These basic finding can eventually be translated into comprehensive strategies to exploit the use of endophytic bacteria to improve plant establishment and biomass production, which can be applied in sustainable agriculture, bioenergy feedstock production on marginal lands, or fight desertification of arid areas.

## Materials and Methods

### Whole-genome shotgun sequencing

Total DNA was isolated from *Enterobacter* sp. 638 as described for *Bacillus subtilis* according to the method of Bron and Venema [85]. Genome sequencing of *Enterobacter* sp. 638 was performed at the Joint Genome Institute (JGI) (Walnut Creek, California, USA) using a combination of three randomly sheared libraries with inserts in the 3kb, 8kb (plasmids) and 40kb (fosmids) size range. All reads were quality assessed and trimmed for vector sequences before being used for assembly. The Paracel Genome Assembler was used to assemble the libraries. Possible misassemblies were corrected; gaps between contigs were closed by editing in Consed (www.phrap.org/), custom primer walks, or PCR amplification.

### Genome analysis and annotation

Putative CoDing Sequences (CDS) were initially identified by the JGI using three different automated annotation softwares: Generation (Oak Ridge National Laboratory), Glimmer [86], [87], and Critica (V1.05) [88]. Another run of CDS identification was performed via the Magnifying Genome (MaGe) annotation platform (http://www.genoscope.cns.fr/agc/mage/) [89] using AMIGene (Annotation of MIcrobial Genes). All CDS identified were manually reviewed, and false CDS were flagged as “artifact”. The remaining CDS were then submitted to automatic functional annotation via BLAST searches against the UniProt databank in order to determine significant homology. Putative genes coding for enzymes were classified with the PRIAM software [90], transmembrane domains were identified by TransMembrane Hidden Markov Model (TMHMM), and signal peptide were predicted using SignalP 3.0, all embedded in the MAGE software [89]. The genome of *Enterobacter* sp. 638 was submitted to the TransportDB [91], [92] website (http://www.membranetransport.org) to identify transporter families by Transporter Automatic Annotation Pipeline tool (TransAAP). Finally, tRNA genes were identified with tRNAscan-SE. Putative orthologs and groups of synteny (which represent the conservation of the chromosomal co-localization between pairs of orthologous genes from different genomes) were computed between *Enterobacter* sp. 638 and all other completed genomes, with a focus on the well known and well annotated genome of the closely related *E. coli* K12. Gene annotations were transferred between *E. coli* K12 and *Enterobacter* sp. 638 for genes sharing 80% identity on 80% of their length, or for genes in synteny (at least 3 genes in synteny between each genome) sharing 70% identity on 80% of their length. Using this method, 2675 genes were annotated for the *Enterobacter* sp. 638 chromosome based on *E. coli* K12. The annotated sequence data are available at http://genome.jgi-psf.org/ent_6/ent_6.home.html, and via GenBank accession numbers CP000653 and CP000654 for the *Enterobacter* sp. 638 chromosome and plasmid pENT638-1, respectively.

### Genomic comparison

Genome comparisons were performed using MaGe [89]. The “PhyloProfile Synteny” program was used to build a Venn diagram displaying the number of homologous genes in related bacteria. Genomic Islands (GI) were identified using the automated “Genomic Islands” tool, followed by a manual curation focusing on several GI properties (Mergeay et al., 2009). These properties include the presence at one extremity of a site-specific recombinase, the preferential insertion of GI at tRNA sites, the presence of flanking insertion sequence elements, a base composition and/or phylogeny which differs from the bulk of the genome, a higher content in hypothetical genes than the neighboring regions, the presence of hot spots for mobile genetic elements (MGEs) including recombinase genes, IS elements, integrase and transposase genes, and the conservation of GI between different unrelated hosts together with their absence in related hosts. A region was considered as a genomic island if at least three criteria were met. Metabolic reconstructions were performed using both the PRIAM software, which is based on the KEGG database, and the MetaCyc/EcoCyc tools embedded into the MAGE platform. The identification of prophages was done using “Prophinder” [93] (http://www.aclame.ulb.ac.be/Tools/Prophinder/). IS Finder (http://www-is.biotoul.fr/) was used for the classification into families of the identified IS elements.

### Induction of *Enterobacter* sp. 638 with sucrose

An *Enterobacter* sp. 638 culture was grown overnight in Schatz-lactate (0.2% w/v) medium [94] at 30°C, 180 rpm until an Optical Density (OD_660nm_) of 0.7 was achieved. Replica cultures were made by 100 fold dilution of the ON culture in Schatz-lactate medium and Schatz-sucrose (0.2% w/v) medium. The cultures were incubated at 30°C, 180 rpm and sampled after 6, 8, 10, 12 and 24 hours for metabolite analysis and total RNA extraction.

### Mass spectrometry

Volatile compounds (including 2-phenylethanol, acetoin, diacetyl and 2,3-butanediol) were extracted using the method described by Romano [95]. At each time point, 1mL sample was mixed with 1mL of ethyl acetate and 1mL of 1M KH_2_PO4. The tube was vortexed briefly, allowed to rest for 1 minute and the upper organic layer was removed for analysis.

Analysis was performed on a VG70S double-focusing, magnetic sector mass spectrometer interfaced to a Hewlett Packard 5890 gas chromatograph. The GC column used was a Restek Carbowax column (30m × 0.25mm ID × 0.25µm df). The helium carrier gas was maintained at 10psi and a split-less injection time of 0.5 minutes was used. The GC started at an initial temperature of 50°C for 3 minutes, ramped 10°C per minute to a final temperature of 250°C and held there for 5 minutes. The spectrometer was scanned from 41–400 amu. Spectra were compared to reference spectra in the NIST 08 Mass Spectral Library.

### Transcriptomic analysis via Quantitative RT–PCR

Total RNA was isolated (RNeasy Midi Kit, Qiagen) after 6, 8, 10, 12 and 24 hours. 16S and 23S rRNA was removed (MICROB*Express*, Ambion) and the remaining RNA (including mRNA) was converted into cDNA (Reverse Transcription kit, Applied Biosystems). Quantitative PCR was performed as previously described [96], using specific primer sets (see Table S1). The housekeeping gene *recA* was used as control for constitutive gene expression. Quantitative PCR reactions were performed in triplicate with SYBR Green (iQ SYBR Green Supermix, Bio-Rad, USA) on an iCycler (Bio-Rad, USA). Data were analyzed by normalization against *recA* levels. Calculation of fold induction was done by comparing gene expression for growth in the presence of sucrose to growth in the presence of lactate.

## Supporting Information

Figure S1 Percentage of genes from a particular COG class depending of their genetic localization: chromosome or plasmid pENT638-1. Legend of the Cog class: D: Cell cycle control, cell division, chromosome partitioning; M, Cell wall/membrane/envelope biogenesis; N, Cell motility; O, Posttranslational modification, protein turnover, chaperones; T, Signal transduction mechanisms; U, Intracellular trafficking, secretion, and vesicular transport; V, Defense mechanisms; W, Extracellular structures; J, Translation, ribosomal structure and biogenesis; K, Transcription; L, Replication, recombination and repair; C, Energy production and conversion; E, Amino acid transport and metabolism; F, Nucleotide transport and metabolism; G, Carbohydrate transport and metabolism; H, Coenzyme transport and metabolism; I, Lipid transport and metabolism; P, Inorganic ion transport and metabolism; Q, Secondary metabolites biosynthesis, transport and catabolism; R, General function prediction only; S, Function unknown.
(0.20 MB TIF)

Figure S2 Distribution of the palindromic repeats on the chromosome of *Enterobacter* sp. 638. Circles display (from the outside): predicted CDSs transcribed in the clockwise and counterclockwise direction, the position of all the palindromic repeats and of the “CCCTCTCCCXX(X)GGGAGAGGG” palindromic repeat found on the *Enterobacter* sp. 638 genome, the GC percent deviation, GC skew. The table on the side shows the variation of XX(X) nucleotide sequences and their cumulative numbers.
(0.63 MB TIF)

Figure S3 Mass Spectrum of the volatile compounds produces by *Enterobacter* sp. 638 grown for 12 hours in Schatz medium supplemented with lactate (4A) or sucrose (4B) as sole carbon source. After analysis of the data using the NIST 08 Mass Spectral Library software and comparison to reference standards, the compounds whose synthesis was induced by the presence of sucrose were identified as the phytohormones acetoin (arrow 1) and 2,3-butanediol (arrow 2).
(0.60 MB TIF)

Table S1 Primer sets used for quantitative PCR.
(0.03 MB DOC)

Table S2 Putative orthologous relationship between the chromosome of *Enterobacter* sp. 638 and the sequenced chromosomes of other members of the *Enterobacteriaceae*.
(0.02 MB PDF)

Table S3 Genomic regions identified on the genome of *Enterobacter* sp. 638. The regions shown in the table are numbered from 1 to 56, and for each one the flanking locus number, the genome coordinates, the size in base pairs and the number of genes that each region contains are indicated. The putative roles of genes within each region are also summarized. A region is defined by a minimum of three consecutive genes that are absent from the *E. coli* K12 genome sequence. The grey shaded rows indicates putative genomic island according to criteria summarized in the columns: *int* (presence of a gene encoding a phage integrase), *tnp* (presence of a gene encoding a transposase), repeat on ext. (presence of repeat at the region extremities), prophage (presence of a prophage according to prediction made by prophinder, tRNA (presence of a flanking tRNA CDS), alternat. codon matrix (the gene are encoding with an alternative matrix of codon compared to the rest of the genome), synteny with K12 (the genes in a particular region are not in synteny compared with *E. coli* K12.
(0.03 MB PDF)

Table S4 Comparison of transporter systems present in *Enterobacter* sp. 638, *Serratia proteamaculans* 568, *E. coli* K12 and O157-H7, *Erwinia carotovorans* SCRI1043, and *Klebsiella pneumoniae* MGH78578 and 342.
(0.06 MB PDF)
