# Supplementary figures and images for: Genome Sequence of the Plant Growth Promoting Endophytic Bacterium Enterobacter sp. 638
Source: PLoS Genet. 2010 May 13;6(5):e1000943. doi: 10.1371/journal.pgen.1000943 (PMC2869309; doi:10.1371/journal.pgen.1000943)

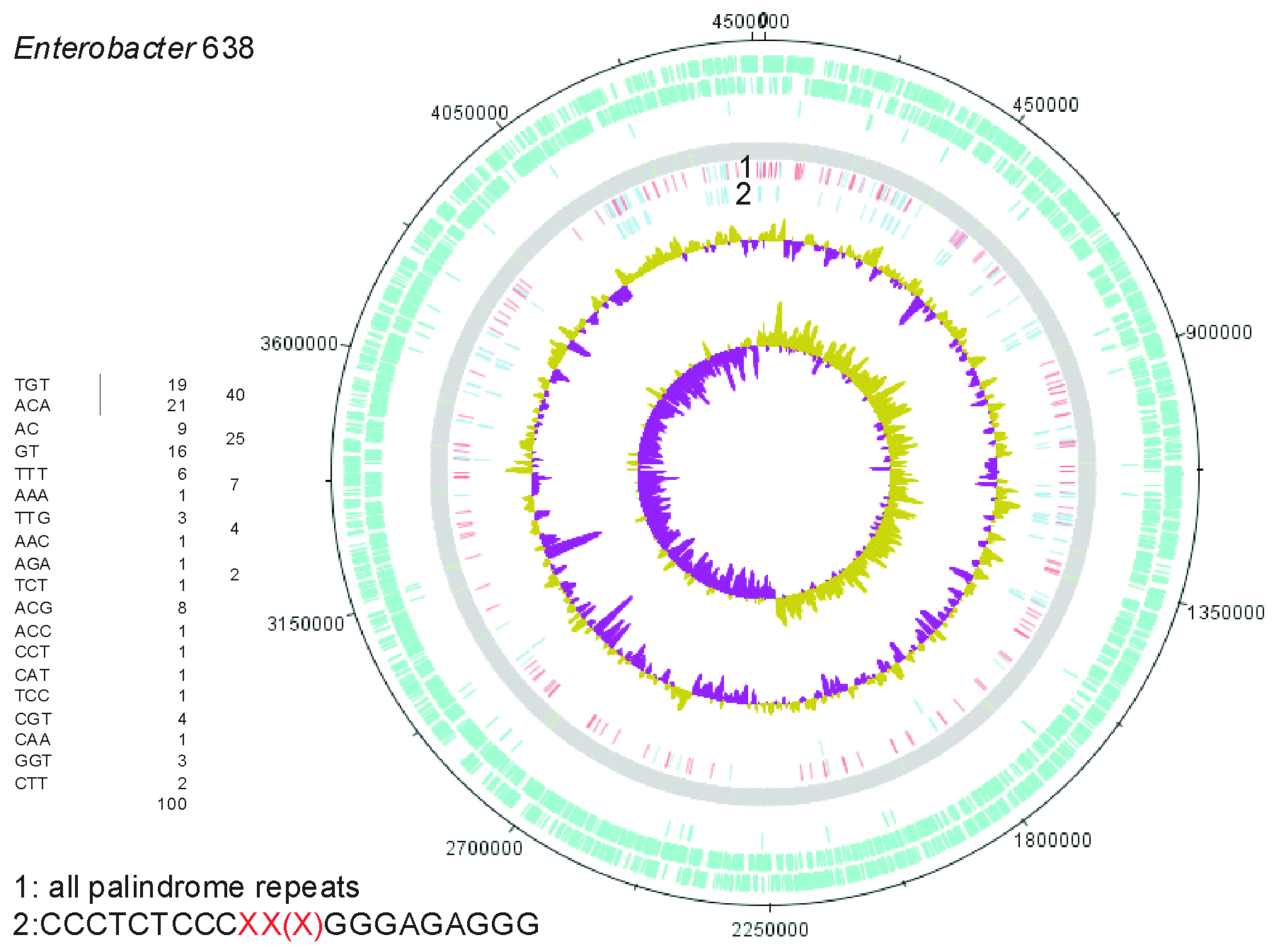

Supplement: Figure S2 — Distribution of the palindromic repeats on the chromosome of Enterobacter sp. 638. Circles display (from the outside): predicted CDSs transcribed in the clockwise and counterclockwise direction, the position of all the palindromic repeats and of the “CCCTCTCCCXX(X)GGGAGAGGG” palindromic repeat found on the Enterobacter sp. 638 genome, the GC percent deviation, GC skew. The table on the side shows the variation of XX(X) nucleotide sequences and their cumulative numbers. (0.63 MB TIF) [file pgen.1000943.s002.tif]

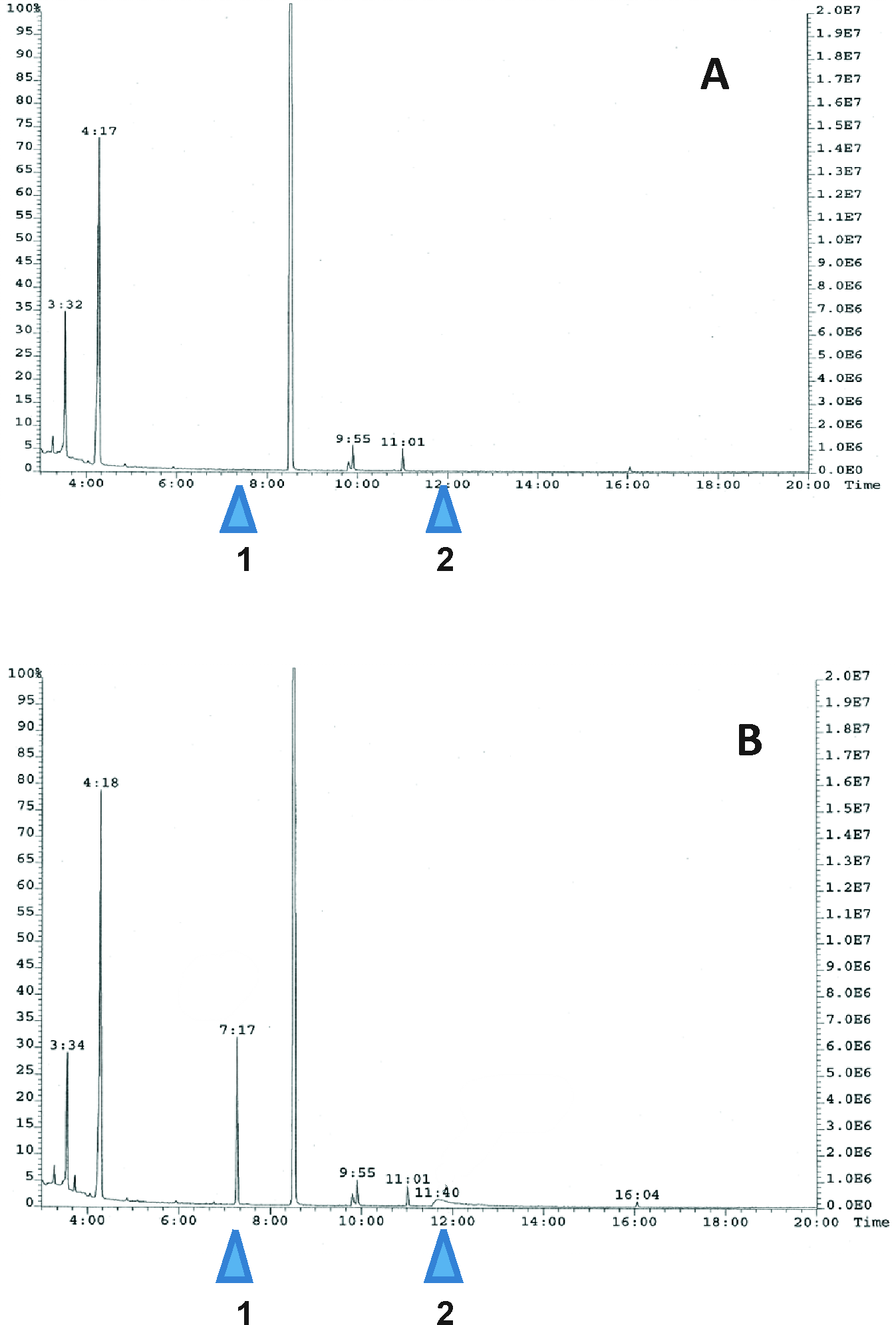

Supplement: Figure S3 — Mass Spectrum of the volatile compounds produces by Enterobacter sp. 638 grown for 12 hours in Schatz medium supplemented with lactate (4A) or sucrose (4B) as sole carbon source. After analysis of the data using the NIST 08 Mass Spectral Library software and comparison to reference standards, the compounds whose synthesis was induced by the presence of sucrose were identified as the phytohormones acetoin (arrow 1) and 2,3-butanediol (arrow 2). (0.60 MB TIF) [file pgen.1000943.s003.tif]
