# Supplementary material for: Genome Sequence of the Plant Growth Promoting Endophytic Bacterium Enterobacter sp. 638
Source: PLoS Genet. 2010 May 13;6(5):e1000943. doi: 10.1371/journal.pgen.1000943 (PMC2869309; doi:10.1371/journal.pgen.1000943)
Supplement: Table S1 — Primer sets used for quantitative PCR. (0.03 MB DOC) [file pgen.1000943.s004.doc]

**Table S-1:** primer sets used for quantitative PCR.

| **Gene identifier** | **Gene-F/R** | **Sequence** | **Tm** |
| --- | --- | --- | --- |
| Ent638_2026 | *budA*-F | GGCGAAATGATTGCCTTCAG | 59 |
| Ent638_2026 | *budA*-R | CCAGGTCATTACTGCGAAAGGT | 59 |
| Ent638_2028 | *budC*-F | TTTGCGGCAGTGGAGAAAG | 59 |
| Ent638_2028 | *budC*-R | TGGCGTGATCGACTCAATTG | 59 |
| Ent638_2022 | *scrB*-F | CGGTACGCACGTGTTGATCT | 58 |
| Ent638_2022 | *scrB*-R | AGTGAAAATCGCCGCTCATC | 56 |
| Ent638_3174 | *RecA*-F | CCACGCTAACCTTACAGG | 56 |
| Ent638_3174 | *RecA*-R | AGTTTACGGGCATACACC | 54 |

The gene identifier indicates the number of the gene used as original identifier on the *Enterobacter* sp. 638 genome sequence.

F or R behind the gene name indicates the orientation of the primers (Forward or reverse, respectively) in function of the orientation of their gene targets.

Tm: melting temperature.
