# Supplementary material for: Genome Sequence of the Plant Growth Promoting Endophytic Bacterium Enterobacter sp. 638
Source: PLoS Genet. 2010 May 13;6(5):e1000943. doi: 10.1371/journal.pgen.1000943 (PMC2869309; doi:10.1371/journal.pgen.1000943)
Supplement: Table S2 — Putative orthologous relationship between the chromosome of Enterobacter sp. 638 and the sequenced chromosomes of other members of the Enterobacteriaceae. (0.02 MB PDF) [file pgen.1000943.s005.pdf]

Enterobacter sp. 638 chromosome Ent638_ NC_009436 584 (4247 CDS)

Putative orthologous relations between two genomes are defined as gene couples satisfying the **bi-directional best hit** (**BBH**) criterion **or** a **blastP alignment threshold**, a minimum of **35%** sequence **identity** on **80%** of the length of the smallest protein. These relations are subsequently used to search for conserved gene clusters, e.g. **synteny groups** (**syntons**) among several bacterial genomes. All possible kinds of chromosomal rearrangements are allowed (inversion, insertion/deletion). A **gap parameter**, representing the maximum number of consecutive genes which are not involved in a synteny group, is set to **five genes**.

limit set: 50% of the genes in synton. In red: PKGDB in black refseq

**QUERY**				**SYNTONS**				**VS**					
**CDS in Syntons**		**BBH**		Synton Nb	**Synton size (gene Nb)**			**BBH**		**CDS in Syntons**		CDS Nb	Replicon Name
Nb	%	Nb	%		Min	Avg	Max	Nb	%	Nb	%		
3415	80.41	3398	80.01	310	1	12.25	198	3398	74.32	3432	75.07	4572	Enterobacter cancerogenus ATCC 35316 NZ_ABWM
3170	74.64	3297	77.63	437	1	8.37	95	3297	60.77	3257	60.04	5425	Klebsiella pneumoniae 342 NC_011283
3135	73.82	3237	76.22	372	1	9.38	115	3237	67.78	3179	66.56	4776	Klebsiella pneumoniae subsp. pneumoniae MGH 78578 NC_009648
3106	73.13	3153	74.24	314	1	10.97	138	3153	63.31	3151	63.27	4980	Citrobacter koseri ATCC BAA-895 NC_009792
3100	72.99	3177	74.81	581	1	6.54	69	3177	49.53	3453	53.84	6414	Escherichia coli O157:H7 str. EC4024 NZ_ABJT
3054	71.91	3126	73.6	425	1	8.24	109	3126	53.87	3179	54.78	5803	Escherichia coli O157:H7 str. TW14588 NZ_ABKY
3037	71.51	3102	73.04	370	1	9.22	109	3102	59.31	3133	59.9	5230	Escherichia coli O157:H7 str. Sakai NC_002695
3025	71.23	3098	72.95	383	1	8.95	110	3098	58.32	3155	59.39	5312	Escherichia coli O157:H7 EDL933 NC_002655
3024	71.2	3074	72.38	362	1	9.3	114	3074	65.46	3058	65.12	4696	Escherichia coli S88 NC_011742
3018	71.06	3072	72.33	370	1	9.03	109	3072	63.66	3028	62.74	4826	Escherichia coli UMN026 NC_011751
3017	71.04	3088	72.71	330	1	9.95	101	3088	61.5	3041	60.57	5021	Escherichia coli UTI89 NC_007946
3014	70.97	3086	72.66	382	1	8.95	107	3086	57.76	3101	58.04	5343	Escherichia coli O157:H7 str. EC4045 NZ_ABHL
3012	70.92	3084	72.62	385	1	8.88	107	3084	58.02	3093	58.19	5315	Escherichia coli O157:H7 str. EC4115 NC_011353
3010	70.87	3047	71.74	364	1	9.13	118	3047	67.07	3018	66.43	4543	Escherichia coli LF82 chromosome LF82_ NC_011993
3004	70.73	3076	72.43	335	1	9.79	105	3076	61.42	3038	60.66	5008	Escherichia coli 042 chromosome EC42_ EC42
3002	70.69	3056	71.96	340	1	9.77	129	3056	67.11	3026	66.45	4554	Escherichia coli O127:H6 str. E2348/69 NC_011601
3000	70.64	3056	71.96	350	1	9.42	111	3056	65.31	2995	64.01	4679	Escherichia coli SE11 NC_011415
2999	70.61	3060	72.05	340	1	9.68	85	3060	57.31	3047	57.07	5339	Escherichia coli CFT073 NC_004431
2997	70.57	3043	71.65	370	1	9.17	105	3043	61.91	3074	62.54	4915	Escherichia coli ED1a NC_011745
2996	70.54	3044	71.67	338	1	9.7	107	3044	64.18	3029	63.86	4743	Escherichia coli SMS-3-5 NC_010498
2988	70.36	3007	70.8	305	1	10.61	99	3007	71.6	2986	71.1	4200	Escherichia coli ATCC 8739 NC_010468
2987	70.33	3035	71.46	352	1	9.4	109	3035	63.72	2993	62.84	4763	Escherichia coli 55989 NC_011748
2978	70.12	3020	71.11	340	1	9.64	109	3020	69.38	2969	68.21	4353	Escherichia coli IAI1 NC_011741
2968	69.88	3041	71.6	415	1	8.14	84	3041	58.26	3055	58.52	5220	Escherichia coli O157:H7 str. EC869 NZ_ABHU
2964	69.79	3012	70.92	310	1	10.23	96	3012	53.87	2951	52.78	5591	Salmonella enterica subsp. enterica serovar Paratyphi B str. SPB7 NC_010102
2957	69.63	3023	71.18	274	1	11.58	152	3023	71.05	2924	68.72	4255	Enterobacter sakazakii ATCC BAA-894 NC_009778
2954	69.55	3003	70.71	337	1	9.61	90	3003	62.52	2952	61.46	4803	Escherichia coli 53638 NZ_AAKB
2948	69.41	2979	70.14	361	1	9.06	99	2979	65.65	2964	65.32	4538	Escherichia coli B REL606 chromosome ECB_ NC_012967
2948	69.41	3004	70.73	321	1	9.93	110	3004	63.26	2951	62.14	4749	Escherichia coli E24377A NC_009801

QUERY				SYNTONS								VS	
CDS in Syntons		BBH		Synton Nb	Synton size (gene Nb)			BBH		CDS in Syntons		CDS Nb	Replicon Name
Nb	%	Nb	%		Min	Avg	Max	Nb	%	Nb	%		
2947	69.39	2990	70.4	315	1	10.14	100	2990	70.75	2950	69.81	4226	Escherichia coli str. K-12 substr. W3110 AC_000091
2940	69.23	2987	70.33	314	1	10.11	98	2987	64.65	2976	64.42	4620	Escherichia coli 536 NC_008253
2938	69.18	2985	70.28	304	1	10.47	100	2985	72.24	2932	70.96	4132	Escherichia coli str. K-12 substr. MG1655 NC_000913
2937	69.15	3020	71.11	398	1	8.36	82	3020	57.61	3009	57.4	5242	Escherichia coli O157:H7 str. EC4076 NZ_ABHQ
2935	69.11	2971	69.96	288	1	10.92	98	2971	67.17	2908	65.75	4423	Salmonella enterica subsp. enterica serovar Typhimurium str. LT2 NC_003197
2934	69.08	3004	70.73	376	1	8.85	103	3004	57.42	3004	57.42	5232	Escherichia coli O157:H7 str. EC4042 NZ_ABHM
2919	68.73	2966	69.84	331	1	9.53	94	2966	69.53	2891	67.77	4266	Escherichia fergusonii ATCC 35469 NC_011740
2918	68.71	3006	70.78	404	1	8.13	99	3006	55.37	2960	54.52	5429	Escherichia coli O157:H7 str. EC4486 NZ_ABHS
2916	68.66	2954	69.55	317	1	9.98	149	2954	67.47	2906	66.38	4378	Escherichia coli HS NC_009800
2916	68.66	2944	69.32	313	1	10.12	97	2944	63.83	2899	62.86	4612	Salmonella enterica subsp. enterica serovar Newport str. SL254 NC_011080
2910	68.52	2974	70.03	357	1	9	89	2974	62.85	2921	61.73	4732	Escherichia coli IAI39 NC_011750
2899	68.26	2943	69.3	298	1	10.47	117	2943	65.2	2869	63.56	4514	Salmonella enterica subsp. enterica serovar Dublin str. CT_02021853 NC_011205
2895	68.17	2971	69.96	375	1	8.72	84	2971	57.11	2958	56.86	5202	Escherichia coli O157:H7 str. EC4206 NZ_ABHK
2890	68.05	2933	69.06	290	1	10.81	98	2933	69.75	2871	68.28	4205	Salmonella enterica subsp. enterica serovar Enteritidis str. NC_011294
2885	67.93	2923	68.83	307	1	10.21	97	2923	62.81	2860	61.45	4654	Salmonella enterica subsp. enterica serovar Virchow str. SL491 NZ_ABFH
2883	67.88	2931	69.01	308	1	10.13	118	2931	63.03	2856	61.42	4650	Salmonella enterica subsp. enterica serovar Heidelberg str. SL476 NC_011083
2877	67.74	2919	68.73	316	1	9.85	98	2919	61.8	2855	60.45	4723	Salmonella enterica subsp. enterica serovar Newport str. SL317 NZ_ABEW
2873	67.65	2897	68.21	260	1	11.78	104	2897	67.09	2845	65.89	4318	Salmonella enterica subsp. enterica serovar Typhi str. Ty2 NC_004631
2872	67.62	2899	68.26	264	1	11.67	104	2899	65.96	2865	65.19	4395	Salmonella enterica subsp. enterica serovar Typhi str. CT18 NC_003198
2869	67.55	2952	69.51	405	1	7.91	79	2952	57.83	2897	56.75	5105	Escherichia coli E22 NZ_AAJV
2867	67.51	2901	68.31	302	1	10.28	97	2901	63.59	2839	62.23	4562	Salmonella enterica subsp. enterica serovar Agona str. SL483 NC_011149
2863	67.41	2910	68.52	293	1	10.59	82	2910	64.67	2843	63.18	4500	Salmonella enterica subsp. enterica serovar Schwarzengrund str. NC_011094
2861	67.37	2901	68.31	301	1	10.29	119	2901	65	2838	63.59	4463	Salmonella enterica subsp. enterica serovar Saintpaul str. SARA23 NZ_ABAM
2860	67.34	2896	68.19	311	1	10.17	99	2896	70.19	2909	70.5	4126	Escherichia coli str. K-12 substr. DH10B NC_010473
2859	67.32	2908	68.47	277	1	10.96	97	2908	65.9	2831	64.15	4413	Salmonella enterica subsp. enterica serovar Choleraesuis str. NC_006905
2855	67.22	2883	67.88	272	1	11.17	156	2883	64.1	2821	62.72	4498	Salmonella enterica subsp. arizonae serovar 62:z4,z23:-- NC_010067
2853	67.18	2929	68.97	382	1	8.28	62	2929	62.27	2888	61.39	4704	Escherichia coli F11 NZ_AAJU

QUERY				SYNTONS								VS	
CDS in Syntons		BBH		Synton Nb	Synton size (gene Nb)			BBH		CDS in Syntons		CDS Nb	Replicon Name
Nb	%	Nb	%		Min	Avg	Max	Nb	%	Nb	%		
2851	67.13	2946	69.37	414	1	7.77	82	2946	56.82	2902	55.97	5185	Escherichia coli O157:H7 str. EC4401 NZ_ABHR
2846	67.01	2900	68.28	389	1	8.1	72	2900	62.95	2842	61.69	4607	Escherichia coli 101-1 NZ_AAMK
2846	67.01	2890	68.05	255	1	11.8	105	2890	70.61	2804	68.51	4093	Salmonella enterica subsp. enterica serovar Paratyphi A str. ATCC NC_006511
2846	67.01	2881	67.84	277	1	11	105	2881	70.65	2798	68.61	4078	Salmonella enterica subsp. enterica serovar Paratyphi A str. NC_011147
2842	66.92	2879	67.79	303	1	10.22	97	2879	63.25	2843	62.46	4552	Salmonella enterica subsp. enterica serovar Kentucky str. CVM29188 NZ_ABAK
2824	66.49	2898	68.24	319	1	9.66	99	2898	65.45	2851	64.39	4428	Escherichia coli APEC O1 NC_008563
2818	66.35	2862	67.39	325	1	9.4	72	2862	63.93	2795	62.43	4477	Salmonella enterica subsp. enterica serovar Hadar str. RI_05P066 NZ_ABFG
2804	66.02	2910	68.52	415	1	7.64	69	2910	57.37	2853	56.25	5072	Escherichia coli O157:H7 str. EC4196 NZ_ABHO
2803	66	2895	68.17	408	1	7.77	79	2895	58.67	2871	58.19	4934	Escherichia coli E110019 NZ_AAJW
2796	65.83	2856	67.25	325	1	9.34	79	2856	60.41	2772	58.63	4728	Salmonella enterica subsp. enterica serovar Weltevreden str. NZ_ABFF
2788	65.65	2809	66.14	294	1	10.19	97	2809	66.34	2740	64.71	4234	Salmonella enterica subsp. enterica serovar Javiana str. NZ_ABEH
2782	65.51	2815	66.28	270	1	11.04	95	2815	71	2753	69.43	3965	Salmonella enterica subsp. enterica serovar Gallinarum str. 287/91 NC_011274
2775	65.34	2836	66.78	351	1	8.61	77	2836	61.63	2760	59.97	4602	Salmonella enterica subsp. enterica serovar 4,[5],12:i:- str. NZ_ABAO
2774	65.32	2821	66.42	312	1	9.54	102	2821	66.86	2756	65.32	4219	Shigella sonnei Ss046 NC_007384
2772	65.27	2829	66.61	319	1	9.41	76	2829	63.27	2750	61.51	4471	Salmonella enterica subsp. enterica serovar Heidelberg str. SL486 NZ_ABEL
2770	65.22	2824	66.49	317	1	9.5	79	2824	62.9	2755	61.36	4490	Salmonella enterica subsp. enterica serovar Schwarzengrund str. NZ_ABEJ
2760	64.99	2863	67.41	389	1	7.9	76	2863	58.07	2783	56.45	4930	Escherichia coli B171 NZ_AAJX
2759	64.96	2867	67.51	417	1	7.35	68	2867	61.68	2781	59.83	4648	Escherichia coli B7A NZ_AAJT
2758	64.94	2799	65.91	353	1	8.51	95	2799	62.63	2734	61.18	4469	Salmonella enterica subsp. enterica serovar Saintpaul str. SARA29 NZ_ABAN
2756	64.89	2868	67.53	425	1	7.38	64	2868	57.3	2813	56.2	5005	Escherichia coli O157:H7 str. EC4113 NZ_ABHP
2743	64.59	2787	65.62	374	1	7.87	81	2787	62.03	2778	61.83	4493	Salmonella enterica subsp. enterica serovar Typhi str. E02-1180 NZ_CAAT
2740	64.52	2851	67.13	432	1	7.2	111	2851	56.8	2785	55.49	5019	Escherichia coli O157:H7 str. EC508 NZ_ABHW
2736	64.42	2787	65.62	317	1	9.36	69	2787	63.51	2720	61.99	4388	Salmonella enterica subsp. enterica serovar Kentucky str. CDC 191 NZ_ABEI
2727	64.21	3016	71.01	482	1	6.32	89	3016	61.66	2753	56.29	4891	Serratia proteamaculans 568 NC_009832
2723	64.12	2856	67.25	423	1	7.23	66	2856	55.74	2761	53.88	5124	Escherichia coli O157:H7 str. EC4501 NZ_ABHT
2718	64	2764	65.08	285	1	10.15	142	2764	67.17	2695	65.49	4115	Shigella flexneri 5 str. 8401 NC_008258
2715	63.93	2814	66.26	615	1	4.86	32	2814	53.42	2798	53.11	5268	Salmonella enterica subsp. enterica serovar Typhi str. E00-7866 NZ_CAAR
2698	63.53	2740	64.52	302	1	9.56	143	2740	65.6	2676	64.07	4177	Shigella flexneri 2a str. 301 NC_004337

QUERY				SYNTONS				VS					
CDS in Syntons		BBH		Synton Nb	Synton size (gene Nb)			BBH		CDS in Syntons		CDS Nb	Replicon Name
Nb	%	Nb	%		Min	Avg	Max	Nb	%	Nb	%		
2687	63.27	2713	63.88	408	1	7.09	54	2713	63.03	2722	63.24	4304	Salmonella enterica subsp. enterica serovar Typhi str. E98-3139 NZ_CAAZ
2680	63.1	2767	65.15	594	1	4.98	25	2767	54.89	2796	55.47	5041	Salmonella enterica subsp. enterica serovar Typhi str. J185 NZ_CAAW
2677	63.03	2721	64.07	295	1	9.74	92	2721	65.82	2664	64.44	4134	Shigella boydii Sb227 NC_007613
2672	62.91	2712	63.86	285	1	9.95	143	2712	66.78	2636	64.91	4061	Shigella flexneri 2a str. 2457T NC_004741
2671	62.89	2741	64.54	325	1	8.94	94	2741	62.49	2642	60.24	4386	Escherichia albertii TW07627 NZ_ABKX
2649	62.37	2685	63.22	295	1	9.57	150	2685	63.24	2640	62.18	4246	Shigella boydii CDC 3083-94 NC_010658
2636	62.07	2855	67.22	466	1	6.32	124	2855	68.5	2670	64.06	4168	Yersinia frederiksenii ATCC 33641 NZ_AALE
2629	61.9	2684	63.2	370	1	7.62	69	2684	60.74	2612	59.11	4419	Shigella dysenteriae 1012 NZ_AAMJ
2562	60.32	2611	61.48	315	1	8.63	71	2611	61.13	2551	59.73	4271	Shigella dysenteriae Sd197 NC_007606
2553	60.11	2795	65.81	375	1	7.25	142	2795	70.26	2521	63.37	3978	Yersinia enterocolitica subsp. enterocolitica 8081 NC_008800
2550	60.04	2744	64.61	803	1	3.57	18	2744	40.69	2724	40.39	6744	Salmonella enterica subsp. enterica serovar Typhi str. M223 NZ_CAAX
2541	59.83	2789	65.67	467	1	6.04	57	2789	71.77	2552	65.67	3886	Yersinia intermedia ATCC 29909 NZ_AALF
2498	58.82	2786	65.6	476	1	5.76	89	2786	62.3	2518	56.31	4472	Pectobacterium atrosepticum SCRI1043 NC_004547
2486	58.54	2676	63.01	471	1	5.95	57	2676	71.38	2527	67.4	3749	Yersinia mollaretii ATCC 43969 NZ_AALD
2440	57.45	2636	62.07	453	1	6.01	67	2636	73.65	2476	69.18	3579	Yersinia bercovieri ATCC 43970 NZ_AALC
2389	56.25	2595	61.1	344	1	7.42	83	2595	61.9	2370	56.54	4192	Yersinia pseudotuberculosis YPIII NC_010465
2383	56.11	2576	60.65	391	1	6.72	83	2576	62.07	2370	57.11	4150	Yersinia pseudotuberculosis PB1/+ NC_010634
2376	55.95	2587	60.91	346	1	7.41	83	2587	66.32	2376	60.91	3901	Yersinia pseudotuberculosis IP 32953 NC_006155
2361	55.59	2574	60.61	345	1	7.3	82	2574	62.42	2342	56.79	4124	Yersinia pseudotuberculosis IP 31758 NC_009708
2356	55.47	2777	65.39	1031	1	2.67	15	2777	37.87	2607	35.55	7333	Salmonella enterica subsp. enterica serovar Typhi str. E98-0664 NZ_CAAU
2334	54.96	2524	59.43	366	1	6.82	60	2524	60.57	2364	56.73	4167	Yersinia pestis Antiqua NC_008150
2332	54.91	2482	58.44	331	1	7.68	69	2482	72.42	2291	66.85	3427	Erwinia tasmaniensis Et1/99 NC_010694
2332	54.91	2518	59.29	348	1	7.15	60	2518	63.25	2344	58.88	3981	Yersinia pestis Nepal516 NC_008149
2311	54.41	2509	59.08	337	1	7.28	59	2509	65.17	2295	59.61	3850	Yersinia pestis Pestoides F NC_009381
2289	53.9	2729	64.26	1016	1	2.61	14	2729	38.5	2500	35.27	7089	Salmonella enterica subsp. enterica serovar Typhi str. E98-2068 NZ_CAAV
2270	53.45	2481	58.42	333	1	7.26	60	2481	63.76	2250	57.83	3891	Yersinia pestis biovar Microtus str. 91001 NC_005810
2263	53.28	2474	58.25	338	1	7.14	60	2474	60.55	2253	55.14	4086	Yersinia pestis KIM NC_004088
2261	53.24	2456	57.83	388	1	6.39	59	2456	58.13	2238	52.97	4225	Yersinia pestis biovar Antiqua str. E1979001 NZ_AAYV
2259	53.19	2462	57.97	386	1	6.42	59	2462	57.85	2238	52.58	4256	Yersinia pestis biovar Antiqua str. B42003004 NZ_AAYU
2251	53	2467	58.09	330	1	7.26	58	2467	63.5	2228	57.35	3885	Yersinia pestis CO92 NC_003143
2250	52.98	2434	57.31	383	1	6.44	59	2434	58.78	2233	53.92	4141	Yersinia pestis biovar Mediaevalis str. K1973002 NZ_AAYT
2250	52.98	2459	57.9	366	1	6.72	58	2459	63.85	2223	57.73	3851	Yersinia pestis CA88-4125 NZ_ABCD
2243	52.81	2443	57.52	383	1	6.42	58	2443	55.57	2229	50.71	4396	Yersinia pestis biovar Orientalis str. MG05-1020 NZ_AAYS
2241	52.77	2427	57.15	392	1	6.27	59	2427	57.95	2225	53.13	4188	Yersinia pestis biovar Antiqua str. UG05-0454 NZ_AAYR

QUERY				SYNTONS								VS	
CDS in Syntons		BBH		Synton Nb	Synton size (gene Nb)			BBH		CDS in Syntons		CDS Nb	Replicon Name
Nb	%	Nb	%		Min	Avg	Max	Nb	%	Nb	%		
2227	52.44	2428	57.17	372	1	6.53	54	2428	57.08	2208	51.9	4254	Yersinia pestis biovar Orientalis str. F1991016 NZ_ABAT
2206	51.94	2396	56.42	352	1	6.63	56	2396	62.53	2179	56.86	3832	Yersinia pestis Angola NC_010159
2205	51.92	2426	57.12	390	1	6.2	59	2426	53.37	2190	48.17	4546	Yersinia pestis biovar Orientalis str. IP275 NZ_AAOS
2199	51.78	2293	53.99	465	1	5.26	47	2293	37.88	2777	45.88	6053	Sodalis glossinidius 'morsitans' chromosome SG NC_007712
