# Supplementary material for: Genome Sequence of the Plant Growth Promoting Endophytic Bacterium Enterobacter sp. 638
Source: PLoS Genet. 2010 May 13;6(5):e1000943. doi: 10.1371/journal.pgen.1000943 (PMC2869309; doi:10.1371/journal.pgen.1000943)
Supplement: Table S3 — Genomic regions identified on the genome of Enterobacter sp. 638. The regions shown in the table are numbered from 1 to 56, and for each one the flanking locus number, the genome coordinates, the size in base pairs and the number of genes that each region contains are indicated. The putative roles of genes within each region are also summarized. A region is defined by a minimum of three consecutive genes that are absent from the E. coli K12 genome sequence. The grey shaded rows indicates putative genomic island according to criteria summarized in the columns: int (presence of a gene encoding a phage integrase), tnp (presence of a gene encoding a transposase), repeat on ext. (presence of repeat at the region extremities), prophage (presence of a prophage according to prediction made by prophinder, tRNA (presence of a flanking tRNA CDS), alternat. codon matrix (the gene are encoding with an alternative matrix of codon compared to the rest of the genome), synteny with K12 (the genes in a particular region are not in synteny compared with E. coli K12. (0.03 MB PDF) [file pgen.1000943.s006.pdf]

Region	From	Ent638_	to	Ent638_	size	ORFs	int	tnp	Repeat on ext.	prophage	tRNA	alternat. codon matrix	Synteny with K12	Synteny with 342	Synteny with 568	Gene content
1	37506	0034	83855	0066	46349	35	-	+	+	-	tRNA-Sec	+	-	+/- (*)	+/- (*)	transporter for sugar uptake (PTS lactose family), Beta-glucosidase (conversion of cellobiose into glucose or glucoside into glucose), filamentous haemagglutinin, transporter (MFS family), Predicted Zn-dependent hydrolases, ORFs of unknown function
2	93676	0074	103614	0086	9938	13	-	-	-	-	-	-	-	-	-	Fimbriae biosynthesis
3	124182	0108	132857	0114	8675	7	-	-	-	-	-	-	-	-	-	Putative membrane-associated metal-dependent hydrolase, Glycosyltransferase
4	166537	0147	169882	0148	3345	3	-	-	-	-	-	-	-	-	-	Hemolysin activation/secretion protein
5	205544	0179	212385	0183	6841	8	-	-	+	-	tRNA-Pro	-	-	-	-	Rhs, peptidoglycan-binding (LysM), several partial duplication
6	332190	283	335579	286	3389	18	-	-	-	-	-	-	-	+	+	Fructokinase, fructose biphoasphate aldolase
7	364148	0317	373015	0321	8867	5	-	-	+	-	tRNA-Phe	-	-	-	-	Nickel chelation for upake or usage as cofactor, Outer membrane autotransporter with Pectin lyase fold/virulence factor (adhesin)
8	436726	0385	441897	0391	5171	7	-	-	-	-	-	-	-	-	-	Regulator, FMN-dependent NADH-azoreductase 2, Protein of unknown function, Antibiotic resistance
9	454627	0401	464073	0410	9446	11	-	-	-	-	-	-	+/-	-	+	Fimbriae biosynthesis for adhesion/virulence, genes duplicated
10	477929	0423	487952	0435	10023	12	-	-	-	-	-	-	-	+	+	Cytochrome, regulator, unknown function, dihydroorotase (peptidase), putative selenocysteine synthase L-seryl-tRNA(Ser) selenium transferase (Pyridoxal phosphate-dependent)
11	523760	0463	573310	0506	49550	43	+	-	-	-	tRNA-Leu	+	-	-	-	Integrase, phage protein, DNA repair (Dnd proteins), plasmid stabilization system, pectate lyase, oligogalacturonate-specific porin (KdgM), protease, possible anti-oxydant, regulators, autotransporter/filamentous haemagglutinin/adhesin, regulator, trancriptionnal regulator involved in virulence, system de secretion, possibly secretion of virulence factor
12	642565	572	648283	576	5718	5	-	-	-	-	-	-	-	+	+/-	Iron-hydroxymate transporter (MFS and ABC family)
13	852653	0750	860429	0756	7776	7	-	-	-	-	tRNA-Asp	-	-	-	-	Regulator, ABC transporter for amino acids
14a	875620	0770	912564	820	59647	50	+	-	+	Phage 1	tRNA-Thr	+	-	-	-	Integrase, phage proteins
14b	912987	821	935267	0837		10					-		-	-	-	Transduction with Phage 1: alpha/beta hydrolase, fimbrial protein, amino acid transporter, methylatransferase, two component sensor/regulator, permease, S-methylmethionine transporter, S-methylmethionine: homocysteine methyltransferase, haemolysin co-regulated protein (HCP), ferric ABC transporter (syntenic with K12), integrase

Region	From	Ent638_	to	Ent638_	size	ORFs	int	tnp	Repeat on ext.	prophage	tRNA	alternat. codon matrix	Synteny with K12	Synteny with 342	Synteny with 568	Gene content
15	1027052	0924	1042473	0937	15421	15	-	-	-	-	+	-	**+/-**	+	-	Regulator, lactose degradation (syntenic with K12), signal transduction (domain EAL), transporter (beta-glucoside PTS family)
16a	1107864	0996	1154361	1055	46497	59	+	-	-	Phage 2	tRNA-Arg	+	**-**	-	-	Phage integrase, phage proteins
16b	1154789	1056	1223024	1114	68235	58					-		**-**	+	+/-	Transduction with Phage 2: Putative TonB-dependent siderophore receptor, phenylalanine transporter, Nucleoside:H+ symporter, Transcriptional regulator (LacI, XRE, TetR, LysR, GntR), permease (MFS family), fimbriae, dihydropteridine reductase, metallo-hydrolase/oxidoreductase, Ferrichrysobactin TonB dependent siderophore receptor, Enterochelin esterase, P-type ATPase transporter, RND transporter, Ribosomal large subunit pseudouridine synthase A, Putative cold-shock DNA-binding domain protein, TonB-dependent receptor, ABC transporter for amino acids, GCN5-related N-acetyltransferase, ABC transporter for chelated iron (SitABCD)
17	1248443	1135	1258304	1143	9861	8	-	-	-	-	-	-	**+/-**	+	+	ABC transporter Ribose uptake, ribose kinase, Methionine metabolism
18	1386002	1260	1392280	1264	6278	5	-	-	-	-	-	-	**-**	+	+	Histidine degradation (*hutIGCUH*)
19	1433737	1306	1438417	1309	4680	5	-	-	-	-	-	-	**-**	+	-	Aldoketo-oxidoreductase, Glycoside hydrolase (family 1), Transporter (PTS lactose/cellobiose family, IIC subunit), Transcriptional regulator (GntR)
20	1441382	1312	1446428	1314	5046	3	-	-	-	-	-	-	**-**	+	+	Alpha-glucosidases (glycosyl hydrolases family 31), Hexuronate transporter, Periplasmic binding protein/LacI transcriptional regulator
21	1472316	1338	1492323	1361	20007	23	+	-	-	Phage 3	tRNA (rybB)	+	**-**	-	-	Putative Fucose 4-O-acetylase and related acetyltransferases, phage proteins, putative TonB-dependent siderophore receptor
22	1533390	1400	1544126	1406	10736	7	-	-	+	-	-	+	**-**	-	-	Crispr associated protein
23	1639354	1484	1691939	1538	52585	29	-	-	+	-	tRNA-Ser	+/-	**-**	+/- (*)	-	Cyclopropane-fatty-acyl-phospholipid synthase, Amine oxidase, transporter (MFS), transcriptional regulator, Glycosyltransferase, Methionine aminopeptidase (MAP) (Peptidase M), arylsulfatase : sulfur metabolism, alternative pyrimidine degradation pathway, autotransporter/Filamentous haemagglutinin/Adhesin (fragments), IS transposase (family IS110), Chloramphenicol acetyltransferase (CAT), alternative pyrimidine degradation pathway
24	1804662	1650	1812852	1661	8190	12	+	-		Phage 4	-	+	**-**	-	- (*)	Phage proteins

Region	From	Ent638_	to	Ent638_	size	ORFs	int	tnp	Repeat on ext.	prophage	tRNA	alternat. codon matrix	Synteny with K12	Synteny with 342	Synteny with 568	Gene content
25	1886255	1737	1892165	1742	5910	6	-	-	-	-	-	-	**-**	+	+	TonB-dependent heme/hemoglobin receptor family protein for iron uptake
26	1929035	1775	1937050	1781	8015	7	-	-	-	-	tRNA-Val	-	**-**	+/- (*)	+/- (*)	Autotransporter for adhesion, ABC transporter system for amino acid/glutamine uptake, Putative metal-dependent RNase, consists of a metallo-beta-lactamase domain and an RNA-binding KH domain, carbonic anhydrase
27	2000083	1841	2001815	1843	1732	4	+	-	-	-	-	+	**-**	-	-	Phage integrase (fragment), incomplete phage inserted into a two component sensor/regulator (RstAB)
28	2015509	1858	2072420	1909	56911	51	-	-	-	-	-	+	**-**	-	-	Chemotaxis/mobility?, Autotransporter adhesin/invasin-like protein (YadA), Antibiotic biosynthesis, RND efflux system nodulation?, RND efflux system drug resistance, Unknown function but small possible legume lectin, beta domain for attachement, MFS transporter, lysophospholipase, coagulase/fibrinolysin, Phage regulator, SOS response
29	2115297	1949	2225046	2051	109749	103	-	-	+	-	-	-	**+/-**	+	+/-	RND transporter, Pectin acetylesterase, Many gene involved in amino acid transport, Many transcriptional regulator, Putative IAA acetyltransferase, sucrose/fructose utilisation with PTS from the beta-glc family, synthesis of acetoin periplasmic disulfide isomerase/thiol-disulphide oxidase (DsbG), depolymerisation of alginates, many transporters and many regulators
30	2260061	2081	2272628	2096	12567	16	-	-	+	-	-	-	**+/-**	+	+/-	Glutamate ABC transporter, Amino acid ABC transporter, Chemiotaxis : aerotaxis
31	2285577	2108	2302826	2119	17249	12	-	-	-	-	-	-	**-**	-	+/- (*)	Virulence proteins SrfA, methionine synthase, Polygalacturonase, pectate lyase (secreted), chondroitin AC/alginate lyase, together with pectate lyase important for colonisation (secreted), putative hydrolase (secreted), Transcriptional regulator, Chemiotaxis : aerotaxis
32a	2405497	2214	2451788	2269	65162	55	+	-	-	Phage 5	-	+	**-**	-	-	Phage, phage integrase
32b	2451959	2270	2470659	2294		24					-		**-**	-	-	Transduction with Phage 6: GCN5-related N-acetyltransferase, Transcriptional regulator (TetR), N-ethylmaleimide reductase, Oxidoreductase, permease/transporter, dehydrogenase, putative intracellular septation protein involved in cell division, hydrolase, membrane spanning TonB, 2-dehydropantoate, Putative drug/metabolite exporter (DMT family),

Region	From	Ent638_	to	Ent638_	size	ORFs	int	tnp	Repeat on ext.	prophage	tRNA	alternat. codon matrix	Synteny with K12	Synteny with 342	Synteny with 568	Gene content
33	2504012	2320	2519110	2329	15098	10	+	-	+	-	-	-	-	+	+	Integrase, nitrate reductase (NasA), nitrate reductase (NasB), nitrate transport (NrtCBA), region flanked by the *nar* operon involved in nitrate reduction and nitrate/nitrite transport
34	2534142	2346	2547263	2356	13121	11	-	-	-	-	-	-	-	-	-	oxidoreductase, Amino acid ABC transporter, purine ribonuclease efflux, trehalase (trehalose degradation), tonB-dependent siderophore
35	2652901	2458	2661174	2464	8273	7	+	-	+	-	-	-	-	-	+	integrase, fimbria/pili (located next to chemotaxis genes and fimbria genes)
36	2706828	2510	2720695	2521	13867	12	-	-	-	-	-	-	-	-	-	Acyl-CoA reductase (LuxC) and Acyl-protein synthetase (LuxE) which are substrat for light production by luciferase, Transketolase, fatty acid biosynthesis
37a	2747355	2553	2783747	2578	36392	25							-	+	-	Transduction with Phage 7: Outer membrane protein N, N-acetylmuramic acid 6-phosphate etherase, Two-component sensor/regulator, Thiamine biosynthesis lipoprotein, Putative NADH:flavin oxidoreductase, Tartrate transporter, anaerobic class I fumarate hydratase, regulators (for cysteine biosynthesis and nitrogen assimilation), P1-type ATPase, Universal stress protein G, transporter (RND), Putative acyltransferases, palmitoyl transferase for Lipid A, shikimate transporter, AMP nucleosidase, Aminopeptidase P, four tRNA-Asn locus, DNA gyrase inhibitor D-alanyl-D-alanine carboxypeptidase
37b	2784850	2579	2824258	2626	39408	49	+	-	-	Phage 6	tRNA-Asn	+	-	-	-	Phage integrase, phage proteins
38	2847062	2647	2851589	2650	4527	4	-	-	-	-	-	-	-	-	-	LPS biosynthesis
39	2902726	2690	2935856	2719	33130	30	-	-	-	-	-	+	-	+/- (*)	+/- (*)	glutathione peroxidase, phosphorilation of lipid, amino acid ABC transporter, diaminobutyrate catabolism, tyrosine kinase, phosphatase
40	3125655	2887	3162212	2916	36557	30	+	-	-	-	tRNA-Arg	+	-	+	-	Putative integrated plasmid: phage integrase, plasmid function, phage integrase, surface reorganisation resulting in increased adherence and increased conjugation frequency
41	3236067	2980	3241398	2983	5331	4	-	+	-	-	-	-	-	-	-	Transposase (IS481), Transporter (PTS Lactose family), Asparaginase, leucyl amidopeptidase
42	3253890	2994	3259362	2997	5472	4	-	-	-	-	-	-	-	+	-	Transcriptional regulator, MFS transporter, beta-xylosidase, Xyloside transporter

Region	From	Ent638_	to	Ent638_	size	ORFs	int	tnp	Repeat on ext.	prophage	tRNA	alternat. codon matrix	Synteny with K12	Synteny with 342	Synteny with 568	Gene content
43a	3375316	3101	3420151	3112	3386099	11	+	-	-	Phage 7	tmRNA	+/-	-	-	-	Phage integrase, endonuclease, phage protein (uncomplete phage)
43b	3386662	3113	3429832	3146	43170	15					-		-	-	-	Transduction with Phage 8: Kinase, Sigma/anti-sigma factor, Putative hemagglutinin/hemolysin protein, Hemagglutinin transporter (outer membrane protein, ABC permease, MFP), putative 2-aminoadipate transaminase, non-haem manganese-containing catalase rpoS-dependent (KatN), Cytochrome bd ubiquinol oxidase, subunit I & II, competence damage-inducible protein A, virulence membrane protein (PagC), Transcriptional regulator (LysR), Short-chain dehydrogenase/reductase, Methyltransferase type 11, putative deaminase/amidohydrolase with metallo-dependent hydrolase domain, putative carbamate kinase, Xanthine/uracil/vitamin C permease, putative DNA-binding transcriptional regulator
44	3460156	3176	3462550	3178	2394	3	-	-	-	-	-	-	-	+	+	ABC transporter
45	3491626	3205	3495685	3208	4059	4	-	-	-	-	-	-	-	+	+/-	ABC transporter involved in Fe^{3+} transport (*EitABCD*)
46	3581959	3279	3586075	3282	4116	4	-	-	-	-	+	-	-	+	+	GCN4-N-acetyltransferase, trancriptionnal regulator, 6-P-beta-glucidase, Transporter (PTS lactose/cellobiose family), regulator lacI-like
47	3590526	3287	3609775	3305	19249	19	+	-	-	-	tRNA-Gly	-	-	+	+/- (*)	IS481, ABC transporter (possibly for sugar with a specialisation in pectin transport) (TogMNAB), Pectin degradation, Oligogalacturonate-specific porin precursor (product of pectin degradation),
48	3688251	3384	3715198	3408	26947	25	-	-	-	-	tRNA-Phe	-	-	-	-	Autransporter with adhesin domain, antioxidant, Molybdenum ABC transporter, Iron ABC-transport protein, periplasmic-binding component, Mechanosensitive ion channel, Chemiotaxis regulator, Autransporter with a Serine-rich
49	3738015	3433	3750557	3442	12542	10	-	-	-	-	-	-	-	+/- (*)	-	Sugar transporter (MFS), Iron compound-binding protein of ABC transporter family, periplasmic component (iron-enterobactin transporter), TonB-
50	3772076	3463	3777014	3469	4938	7	-	-	-	-	-	-	-	+	-	Urease (*ureDABCEFG*)
51a	3783633	3475	3814474	3514	30841	39	+	-	-	Phage 8	tRNA-Met	+	-	-	-(*)	Phage integrase, phage proteins (conserved in *K. pneumoniae*, *E. coli* UTI89)
51b	3814471	3516	3832226	3530	17755	24							-	-	-	Transduction with Phage 9: Phosphatidylglycerol membrane-oligosaccharide glycerophosphotransferase, Transcriptional regulators (LysR, TetR, XRE), Metallo hydrolase,
52	4069288	3771	4076336	3779	7048	9	-	-	-	-	-	-	-	+	-	Malonate (*mdc* genes), Malonate transporter (family of auxin efflux carrier) (MdcF)

Region	From	Ent638_	to	Ent638_	size	ORFs	int	tnp	Repeat on ext.	prophage	tRNA	alternat. codon matrix	Synteny with K12	Synteny with 342	Synteny with 568	Gene content
53	4192604	3882	4212209	3905	19605	24	-	-	-	-	-	-	-	-	+ (*)	Fatty acid biosynthesis
54	4255568	3936	4269242	3944	13674	9	-	-	-	-	-	-	-	+	-	Cellulose biosynthesis *(bcsZDCBA)*
55	4294762	3964	4298896	3966	4134	3	-	-	-	-	-	-	-	+	+	Transporter (Beta-glucoside PTS family)
56	4425327	4070	4437770	4081	12443	12	-	-	-	-	-	-	-	+	- (*)	Ribose ABC transporter, raffinose operon (transport/utilisation)
						495										

The coordinate given are those of the genes, not those of the repeat from phage

organism used for the comparison: K. pneumoniae MGH78578, E. coli K12, O157-H7, UTI89, C. koseri BAA-895

Compared with 568 and 342, K12 and 638 have the operons:0231-0234 porins and lipoproteins;
