# Supplementary material for: Genome Sequence of the Plant Growth Promoting Endophytic Bacterium Enterobacter sp. 638
Source: PLoS Genet. 2010 May 13;6(5):e1000943. doi: 10.1371/journal.pgen.1000943 (PMC2869309; doi:10.1371/journal.pgen.1000943)
Supplement: Table S4 — Comparison of transporter systems present in Enterobacter sp. 638, Serratia proteamaculans 568, E. coli K12 and O157-H7, Erwinia carotovorans SCRI1043, and Klebsiella pneumoniae MGH78578 and 342. (0.06 MB PDF) [file pgen.1000943.s007.pdf]

				E. coli		*E. carotovora*	*K. pneumoniae*	
		**Sprot568**	**Ent638**	**K12**	**O157-H7**	**SCRI1043**	**MGH78578**	**342**
**1.A. α-Type channels**								
The Voltage-gated Ion Channel (VIC) Superfamily	1.A.1	1	2	1	1	0	1	1
The Major Intrinsic Protein (MIP) Family	1.A.8	2	2	2	2	1	5	4
The Ammonia Transporter Channel (Amt) Family	1.A.11	1	1	1	1	1	1	1
The Large Conductance Mechanosensitive Ion Channel (MscL) Family	1.A.22	1	1	1	1	1	1	1
The Small Conductance Mechanosensitive Ion Channel (MscS) Family	1.A.23	6	7	6	6	4	7	7
The Urea Transporter (UT) Family	1.A.28	0	0	0	0	0	0	0
The CorA Metal Ion Transporter (MIT) Family	1.A.35	4	2	2	3	2	3	3
	total	15	15	13	14	9	18	17
**2.A. Porters (uniporters, symporters, antiporters)**								
The Major Facilitator Superfamily (MFS)	2.A.1	114	81	70	76	64	119	128
The Glycoside-Pentoside-Hexuronide (GPH):Cation Symporter Family	2.A.2	1	5	6	6	3	8	9
The Amino Acid-Polyamine-Organocation (APC) Family	2.A.3	21	12	22	21	11	20	22
The Cation Diffusion Facilitator (CDF) Family	2.A.4	3	2	2	2	2	5	5
The Zinc (Zn2+)-Iron (Fe2+) Permease (ZIP) Family	2.A.5	1	1	0	0	0	1	1
The Resistance-Nodulation-Cell Division (RND) Superfamily	2.A.6	14	14	9	12	9	14	15
The Drug/Metabolite Transporter (DMT) Superfamily	2.A.7	26	19	16	16	19	25	28
The Gluconate:H+ Symporter (GntP) Family	2.A.8	6	2	7	4	3	4	6
The Cytochrome Oxidase Biogenesis (Oxa1) Family	2.A.9	1	1	1	1	1	1	1
The 2-Keto-3-Deoxygluconate Transporter (KDGT) Family	2.A.10	0	1	1	1	1	1	1
The Citrate-Mg2+:H+ (CitM) Citrate-Ca2+:H+ (CitH) Symporter (CitMHS) Family	2.A.11	0	0	0	0	2	0	0
The ATP:ADP Antiporter (AAA) Family	2.A.12	0	0	0	0	0	0	0
The C4-Dicarboxylate Uptake (Dcu) Family	2.A.13	2	2	2	2	2	2	2
The Lactate Permease (LctP) Family	2.A.14	1	1	2	1	1	1	1
The Betaine/Carnitine/Choline Transporter (BCCT) Family	2.A.15	2	0	3	3	1	3	2
The Telurite-resistance/Dicarboxylate Transporter (TDT) Family	2.A.16	1	1	1	1	0	1	1
The Proton-dependent Oligopeptide Transporter (POT) Family	2.A.17	4	2	4	4	1	6	5
The Ca2+:Cation Antiporter (CaCA) Family	2.A.19	2	2	2	2	2	2	2
The Inorganic Phosphate Transporter (PiT) Family	2.A.20	2	1	2	2	1	1	1
The Solute:Sodium Symporter (SSS) Family	2.A.21	4	3	4	4	4	4	3
The Dicarboxylate/Amino Acid:Cation (Na+ or H+) Symporter (DAACS) Family	2.A.23	4	5	3	5	6	5	5
The 2-Hydroxycarboxylate Transporter (2-HCT) Family	2.A.24	2	1	0	0	2	2	3
The Alanine or Glycine:Cation Symporter (AGCS) Family	2.A.25	1	1	1	1	0	1	1
The Branched Chain Amino Acid:Cation Symporter (LIVCS) Family	2.A.26	2	1	1	1	1	1	1
The Glutamate:Na+ Symporter (ESS) Family	2.A.27	1	0	1	1	0	1	1
The Bile Acid:Na+ Symporter (BASS) Family	2.A.28	3	2	1	1	2	2	2

			E. coli		*E. carotovora*	*K. pneumoniae*	
		**Sprot568**	**Ent638**	**K12**	**O157-H7**	**SCRI1043**	**MGH78578**	**342**

Family	TC	Sprot568	Ent638	K12	O157-H7	SCRI1043	MGH78578	342
The NhaA Na+:H+ Antiporter (NhaA) Family	2.A.33	1	1	1	1	1	2	1
The NhaB Na+:H+ Antiporter (NhaB) Family	2.A.34	1	1	1	1	1	1	1
The NhaC Na:H Antiporter (NhaC) Family	2.A.35	1	0	0	0	0	0	0
The Monovalent Cation:Proton Antiporter-1 (CPA1) Family	2.A.36	2	2	2	2	1	3	3
The Monovalent Cation:Proton Antiporter-2 (CPA2) Family	2.A.37	4	3	3	3	2	3	3
The K+ Transporter (Trk) Family	2.A.38	2	1	2	1	1	1	1
The K Transporter (Trk) Family	2.A.39	2	0	2	2	2	3	4
The Nucleobase:Cation Symporter-2 (NCS2) Family	2.A.40	6	5	10	11	4	7	7
The Concentrative Nucleoside Transporter (CNT) Family	2.A.41	4	2	3	3	3	3	2
The Hydroxy/Aromatic Amino Acid Permease (HAAAP) Family	2.A.42	5	5	8	8	3	7	7
The Formate-Nitrite Transporter (FNT) Family	2.A.44	3	3	4	4	2	2	2
The Arsenite-Antimonite (ArsB) Efflux Family	2.A.45	1	1	2	1	1	2	2
The Benzoate:H+ Symporter (BenE) Family	2.A.46	1	1	1	1	1	1	1
The Divalent Anion:Na+ Symporter (DASS) Family	2.A.47	4	4	5	5	4	6	8
The Chloride Carrier/Channel (ClC) Family	2.A.49	3	3	3	3	0	4	4
The Chromate Ion Transporter (CHR) Family	2.A.51	2	0	0	0	0	1	1
The Ni2+-Co2+ Transporter (NiCoT) Family	2.A.52	2	3	0	0	1	3	3
The Sulfate Permease (SulP) Family	2.A.53	4	2	1	1	2	4	3
The Metal Ion (Mn2+-iron) Transporter (Nramp) Family	2.A.55	2	1	1	1	1	1	2
The Tripartite ATP-independent Periplasmic Transporter (TRAP-T) Family	2.A.56	5	4	3	0	3	0	0
The Phosphate:Na+ Symporter (PNaS) Family	2.A.58	1	1	1	1	1	1	2
The Arsenical Resistance-3 (ACR3) Family	2.A.59	0	0	0	0	0	0	0
The C4-dicarboxylate Uptake C (DcuC) Family	2.A.61	1	2	2	2	1	1	1
The Monovalent Cation (K+ or Na+):Proton Antiporter-3 (CPA3) Family	2.A.63	0	0	0	0	0	0	0
The Twin Arginine Targeting (Tat) Family	2.A.64	4	4	4	4	4	4	4
The Multidrug/Oligosaccharidyl-lipid/Polysaccharide (MOP) Flippase Superfamily	2.A.66	9	8	8	8	5	6	4
The Oligopeptide Transporter (OPT) Family	2.A.67	1	0	0	0	0	0	0
The p-Aminobenzoyl-glutamate Transporter (AbgT) Family	2.A.68	1	1	1	2	0	1	1
The Auxin Efflux Carrier (AEC) Family	2.A.69	1	1	1	1	2	1	3
The Malonate:Na+ Symporter (MSS) Family	2.A.70	0	0	0	0	0	0	0
The K+ Uptake Permease (KUP) Family	2.A.72	2	1	1	1	1	1	1
The Short Chain Fatty Acid Uptake (AtoE) Family	2.A.73	0	0	1	0	0	0	0
The L-Lysine Exporter (LysE) Family	2.A.75	1	1	1	1	1	1	1
The Resistance to Homoserine/Threonine (RhtB) Family	2.A.76	9	4	5	5	11	7	9
The Branched Chain Amino Acid Exporter (LIV-E) Family	2.A.78	1	2	1	1	2	3	2
The Threonine/Serine Exporter (ThrE) Family	2.A.79	1	1	1	0	1	1	1
The Tricarboxylate Transporter (TTT) Family	2.A.80	3	0	0	0	0	0	0

			E. coli		*E. carotovora*	*K. pneumoniae*	
		**Sprot568**	**Ent638**	**K12**	**O157-H7**	**SCRI1043**	**MGH78578**	**342**
The Aspartate:Alanine Exchanger (AAE) Family	2.A.81	2	2	1	0	2	2	2
The Aromatic Acid Exporter (ArAE) Family	2.A.85	5	5	3	3	0	6	8
The Autoinducer-2 Exporter (AI-2E) Family (Formerly the PerM Family, TC #9.B.22)	2.A.86	4	6	0	0	0	0	0
The Vacuolar Iron Transporter (VIT) Family	2.A.89	0	0	0	0	0	0	0
	total	319	241	244	244	202	319	340
**3.A. P-P-bond-hydrolysis-driven transporters**								
The ATP-binding Cassette (ABC) Superfamily	3.A.1	354	295	210	239	358	386	422
The H^+- or Na^+-translocating F-type, V-type and A-type ATPase (F-ATPase) Superfamily	3.A.2	9	9	9	9	9	9	9
The P-type ATPase (P-ATPase) Superfamily	3.A.3	7	8	6	6	6	9	10
The Arsenite-Antimonite (ArsAB) Efflux Family	3.4.4	0	0	0	0	0	1	2
The General Secretory Pathway (Sec) Family	3.A.5	7	6	0	0	0	3	3
The H+-translocating Pyrophosphatase (H+-PPase) Family	3.A.10	0	0	0	0	0	0	0
The Septal DNA Translocator (S-DNA-T) Family	3.A.12	1	1	0	0	0	0	0
	total	378	319	225	254	373	408	446
**4.A. Phosphotransfer-driven group translocators**								
	4.A	45	41	50	63	45	84	78
**9.A. Recognized transporters of unknown biochemical mechanism**								
The MerTP Mercuric Ion (Hg^{2+}) Permease (MerTP) Family	9.A.2	0	0	0	0	0	0	0
The YggT or Fanciful K^+ Uptake-B (FkuB; YggT) Family	9.A.4	1	1	0	0	0	0	0
The Ferrous Iron Uptake (FeoB) Family	9.A.8	1	2	1	1	0	1	1
The Iron/Lead Transporter (ILT) Superfamily	9.A.10	1	1	0	0	0	0	0
The Iron/Lead Transporter (ILT) Superfamily	9.A.18	0	1	1	1	0	1	1
The Mg2 Transporter-E (MgtE) Family	9.A.19	2	2	0	0	1	2	2
The Ethanolamine Facilitator (EAF) Family	9.A.28	1	0	0	0	0	0	0
The Putative 4-Toluene Sulfonate Uptake Permease (TSUP) Family	9.A.29	2	1	0	0	0	0	0
The Tellurium Ion Resistance (TerC) Family	9.A.30	4	4	0	0	0	0	0
The Pyocin R2 Phage P2 Tail Fiber Protein (Pyocin R2) Family	9.A.33	1	1	0	0	0	0	0
The HlyC/CorC (HCC) Family	9.A.40	4	2	0	0	0	0	0
The Capsular Polysaccharide Exporter (CPS-E) Family	9.A.41	0	0	0	0	0	0	0
	total	17	15	2	2	1	4	4
	**TOTAL**	**774**	**631**	**534**	**577**	**630**	**833**	**885**
	%	15.4	14.4	12.9	10.9	14.1	16.1	15.3

sources: (1) http://www.membranetransport.org/ and (2) http://www.tcdb.org/
